# Impact of Simian Immunodeficiency Virus Infection on Chimpanzee Population Dynamics

**DOI:** 10.1371/journal.ppat.1001116

**Published:** 2010-09-23

**Authors:** Rebecca S. Rudicell, James Holland Jones, Emily E. Wroblewski, Gerald H. Learn, Yingying Li, Joel D. Robertson, Elizabeth Greengrass, Falk Grossmann, Shadrack Kamenya, Lilian Pintea, Deus C. Mjungu, Elizabeth V. Lonsdorf, Anna Mosser, Clarence Lehman, D. Anthony Collins, Brandon F. Keele, Jane Goodall, Beatrice H. Hahn, Anne E. Pusey, Michael L. Wilson

**Affiliations:** 1 Department of Microbiology, University of Alabama at Birmingham, Birmingham, Alabama, United States of America; 2 Department of Anthropology, Stanford University, Stanford, California, United States of America; 3 Woods Institute for the Environment, Stanford University, Stanford, California, United States of America; 4 Department of Ecology, Evolution and Behavior, University of Minnesota, St. Paul, Minnesota, United States of America; 5 Department of Medicine, University of Alabama at Birmingham, Birmingham, Alabama, United States of America; 6 Fauna and Flora International, Monrovia, Liberia; 7 Africa Program, Wildlife Conservation Society, Bronx, New York, United States of America; 8 Gombe Stream Research Centre, The Jane Goodall Institute, Kigoma, Tanzania; 9 The Jane Goodall Institute, Arlington, Virginia, United States of America; 10 Lester E. Fisher Center for the Study and Conservation of Apes, Lincoln Park Zoo, Chicago, Illinois, United States of America; 11 The AIDS and Cancer Virus Program, SAIC-Frederick Inc., National Cancer Institute-Frederick, Frederick, Maryland, United States of America; 12 Department of Evolutionary Anthropology, Duke University, Durham, North Carolina, United States of America; 13 Department of Anthropology, University of Minnesota, Minneapolis, Minnesota, United States of America; Harvard University, United States of America

## Abstract

Like human immunodeficiency virus type 1 (HIV-1), simian immunodeficiency virus of chimpanzees (SIVcpz) can cause CD4+ T cell loss and premature death. Here, we used molecular surveillance tools and mathematical modeling to estimate the impact of SIVcpz infection on chimpanzee population dynamics. Habituated (Mitumba and Kasekela) and non-habituated (Kalande) chimpanzees were studied in Gombe National Park, Tanzania. Ape population sizes were determined from demographic records (Mitumba and Kasekela) or individual sightings and genotyping (Kalande), while SIVcpz prevalence rates were monitored using non-invasive methods. Between 2002–2009, the Mitumba and Kasekela communities experienced mean annual growth rates of 1.9% and 2.4%, respectively, while Kalande chimpanzees suffered a significant decline, with a mean growth rate of −6.5% to −7.4%, depending on population estimates. A rapid decline in Kalande was first noted in the 1990s and originally attributed to poaching and reduced food sources. However, between 2002–2009, we found a mean SIVcpz prevalence in Kalande of 46.1%, which was almost four times higher than the prevalence in Mitumba (12.7%) and Kasekela (12.1%). To explore whether SIVcpz contributed to the Kalande decline, we used empirically determined SIVcpz transmission probabilities as well as chimpanzee mortality, mating and migration data to model the effect of viral pathogenicity on chimpanzee population growth. Deterministic calculations indicated that a prevalence of greater than 3.4% would result in negative growth and eventual population extinction, even using conservative mortality estimates. However, stochastic models revealed that in representative populations, SIVcpz, and not its host species, frequently went extinct. High SIVcpz transmission probability and excess mortality reduced population persistence, while intercommunity migration often rescued infected communities, even when immigrating females had a chance of being SIVcpz infected. Together, these results suggest that the decline of the Kalande community was caused, at least in part, by high levels of SIVcpz infection. However, population extinction is not an inevitable consequence of SIVcpz infection, but depends on additional variables, such as migration, that promote survival. These findings are consistent with the uneven distribution of SIVcpz throughout central Africa and explain how chimpanzees in Gombe and elsewhere can be at equipoise with this pathogen.

## Author Summary

It is now well established that human immunodeficiency virus type 1 (HIV-1), which causes acquired immune deficiency syndrome (AIDS), emerged following cross-species transmission of a simian immunodeficiency virus that infects chimpanzees (SIVcpz). Although SIVcpz was originally believed not to be pathogenic, a recent study conducted in Gombe National Park, Tanzania, found that infected chimpanzees can develop AIDS-like symptoms and have a high mortality. To examine the impact of SIVcpz on chimpanzee survival, we examined and then modeled the effect of virus infection on chimpanzee population growth. We studied three communities, including the little-studied Kalande community, located in the south of the park. We found that 46% of Kalande apes were infected with SIVcpz, compared to less than 13% of the other communities. Interestingly, the communities with lower infection rates grew, while Kalande suffered a catastrophic decline. Mathematical modeling indicated that chimpanzee populations infected with SIVcpz are likely to decline, although intercommunity migration counteracted this negative effect, allowing many simulated populations to persist. These results suggest that SIVcpz infection, together with other variables, contributed to the Kalande population decline. However, population extinction is not a necessary consequence of SIVcpz infection, but can be counteracted by other factors, such as migration, that promote population survival. These findings are consistent with the uneven distribution of SIVcpz across Africa and may explain how chimpanzees as a species have survived this pathogen.

## Introduction

Until recently, simian immunodeficiency virus (SIVcpz) of chimpanzees (*Pan troglodytes*), the immediate precursor to human immunodeficiency virus type 1 (HIV-1), was assumed to be non-pathogenic in its natural host [1]. However, a long-term natural history study of infected apes in Gombe National Park revealed that SIVcpz is quite pathogenic, causing CD4+ T cell depletion, lymphatic tissue destruction and premature death [2]. Chimpanzees are already highly endangered and face severe pressure from hunting, habitat destruction, and other diseases [3]–[5]. In this study, we thus asked what additional impact SIVcpz pathogenicity may have on chimpanzee survival at the population level.

Most primate species naturally infected with SIV appear not to develop immunodeficiency, although only African green monkeys (*Chlorocebus* spp.) and sooty mangabeys (*Cercocebus atys*) have been studied in detail [1]. In the latter species, SIV infection is common and widespread throughout the natural habitat [6]–[9]. This is not true for wild-living chimpanzees, where extensive sampling across Africa has shown a rather uneven distribution of SIVcpz, with high prevalence rates in some communities and rare or absent infection in others [10]–[12]. Within the range of the eastern chimpanzee (*Pan troglodytes schweinfurthii*), SIVcpz has been documented at several locations in the Democratic Republic of Congo [12], [13]; however, extensive molecular epidemiological studies of chimpanzee communities in Uganda and Rwanda failed to detect evidence of infection [10], [14]. Similarly, in Tanzania SIVcpz was found in Gombe, but not in Mahale Mountains National Park [10], [14]. The reasons for this uneven distribution remain unclear; however, one possibility is that infected populations go extinct as a consequence of SIVcpz infection, with habitat subsequently colonized by chimpanzees from uninfected populations.

**Figure 1:**
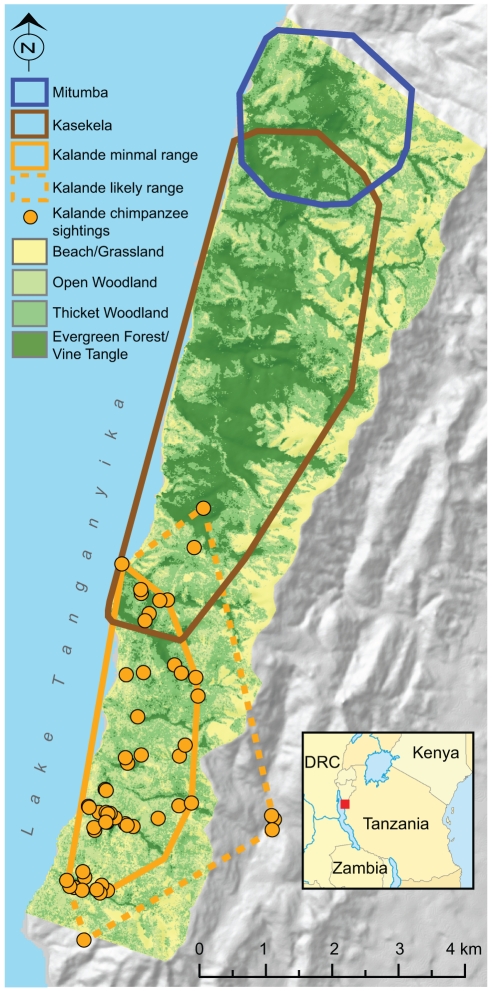
Map of Gombe National Park, Tanzania. The 2007 ranges of the habituated Kasekela (brown) and Mitumba (blue) communities are shown in relation to minimal (solid lines) and likely (broken lines) ranges of the unhabituated Kalande (orange) community. The inset depicts the location of Gombe within Tanzania. Yellow circles indicate observed locations (2002–2009) of Kalande chimpanzees (within the park) and their nests (outside the park). Vegetation cover is colored based on classification from remote sensing as beach/grassland (yellow), open woodland (light green), thicket woodland (medium green), or evergreen forest/vine tangle (dark green).

To examine whether SIVcpz has a negative impact on chimpanzee population growth, we obtained detailed demographic and prevalence data from Gombe National Park, Tanzania. Gombe is home to three chimpanzee communities (Figure 1) and is the only site where habituated chimpanzees are endemically infected with SIVcpz, thus making it the only location where these studies could be conducted. Observational studies of the Kasekela and Mitumba chimpanzees began in 1960 and 1985, and both communities were habituated to close-range observation by the mid-1960s and mid-1990s, respectively [15]. The Kalande community has not been habituated, but has been continuously monitored since 1999 [16]. Non-invasive SIVcpz surveys were initiated in 2000, but did not include sufficiently large numbers of individuals until 2002 [10]. We thus examined data for the 8-year time period for which we have both demographic and SIVcpz prevalence data (2002–2009). This represented a sufficiently long timescale to document both new infections and deaths of infected individuals. To gain a longer-term perspective on demographic patterns, we also examined demographic data going back to 1998, the earliest year for which reliable population size estimates for all three communities are available.

In a previous study, we reported the molecular epidemiology of SIVcpz infection in Kasekela and Mitumba [2]. In the present study, we focused on the unhabituated Kalande community, for three reasons. First, an initial survey conducted in 2002 showed that Kalande chimpanzees exhibited a high SIVcpz prevalence [10]. Second, molecular epidemiological evidence suggested that Kalande played a role in the spread of the SIVcpz infection in Gombe [2]. Finally, Kalande appeared to have undergone a substantial population decline [15]. From the late 1960s through the early 1980s, Kalande was likely comparable in size to the Kasekela community, based on the number of males (7–9 adult males in Kalande compared to 7–8 in Kasekela) and the size of territory that each community controlled [17]. By the late 1990s, however, Kasekela chimpanzees were traveling deep into former Kalande territory. The fact that they encountered little resistance suggested that the Kalande community had undergone a decline, which was confirmed by subsequent population surveys [16], [18]. Concern that the Kalande community was declining prompted a survey in 1998, followed by regular monitoring starting in 1999. Initially, the decline was attributed to human impacts, including habitat loss and poaching [15], [18]. However, an unusually high SIVcpz prevalence in Kalande [10], combined with the now recognized excess mortality associated with this infection [2], suggested that SIVcpz might have contributed to the decline of this community.

Following up on these earlier observations, we have in the present study examined the decline of the Kalande chimpanzees in greater detail. First, we examined whether their decline continued in more recent years, by estimating their annual population size using a combination of nest transect data, visual observations, and non-invasively collected genetic data to infer kinship relations. Second, we used microsatellite data to examine kin relationships of presumed Kalande emigrants. Third, we constructed viral phylogenies to examine the role of Kalande chimpanzees in inter-community transmission of SIVcpz. Fourth, we tested whether habitat loss, rather than disease, was responsible for Kalande's most recent decline by compiling data from vegetation plots and comparing the food abundance in the ranges of each community. Finally, we used empirically determined demographic parameters as well as deterministic and stochastic mathematical models to gain a more general understanding of the impact of SIVcpz associated excess mortality on chimpanzee population size. Collectively, our studies confirmed that the Kalande community has experienced a catastrophic population decline and suggested that this decline was caused, at least in part, by high levels of SIVcpz infection. However, we also found that population extinction is not an inevitable consequence of SIVcpz infection. Stochastic modeling revealed that intercommunity migration can counteract the negative effects of SIVcpz and rescue declining populations. Testing various realistic conditions, we found that SIVcpz frequently went extinct rather than its primate host. These results have important implications for chimpanzee conservation.

## Results

### SIVcpz Prevalence in Gombe

**Figure 2:**
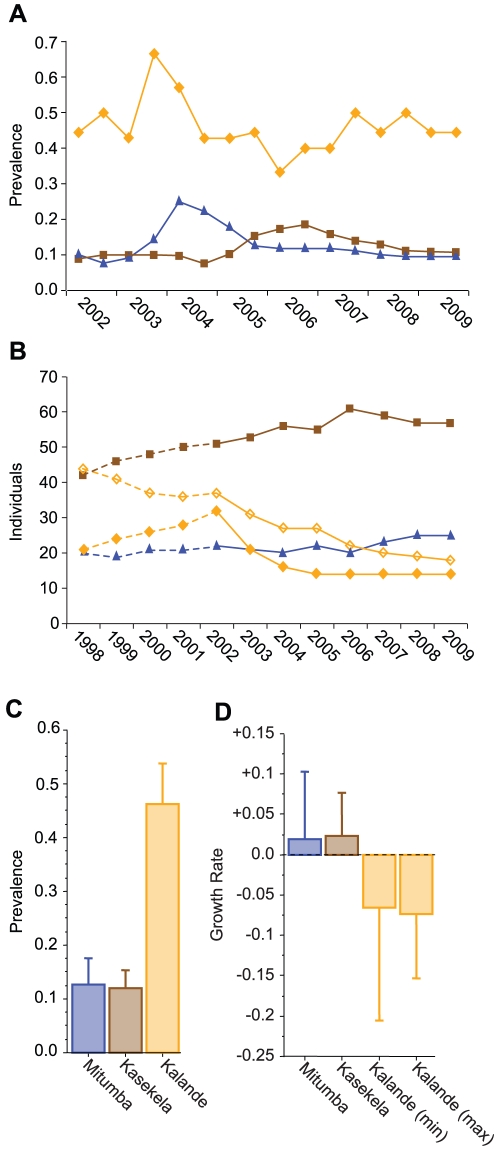
SIVcpz prevalence, population sizes, and median population growth rates for the Gombe communities. Data are color coded according to community (Mitumba: blue; Kasekela: brown; Kalande: orange). (A) Proportion of individuals infected with SIVcpz in the three Gombe communities, as determined by biannual sampling between 2002 and 2009 (based on Figure S1). (B) Population size of the three Gombe communities (including minimum and maximum estimates for Kalande) between 1998 and 2009. Dotted and solid lines connect data points before and after the start of regular SIVcpz sampling in 2002, respectively. (C, D) Mean biannual prevalence (C) and annual growth rates (D) in each Gombe community from 2002–2009 (error bars indicate standard deviations).

Noninvasive SIVcpz testing was initiated in Gombe in 2000, after the first infected chimpanzee (Ch-006) was identified [19]. Subsequent studies documented additional infections and showed that all three communities harbored SIVcpz [10]. By 2001, many Kasekela and Mitumba chimpanzees were sampled at least once every year [2]. Sampling in Kalande was more sporadic due to the non-habituated nature of this community (Figure S1), but over the past eight years 341 fecal samples have been collected from 26 different individuals (median 4.5 samples per individual; range 1 to 75; Tables 1 and S1). Five resident Kalande chimpanzees were followed continuously over six years (Figure S1), one of whom (Ch-100) became newly infected during the course of the study (Table S1). Like in Mitumba and Kasekela, SIVcpz infection in Kalande was determined non-invasively, by documenting virus specific antibodies and/or nucleic acids in fecal samples [2], [10]. The number of sampled individuals was determined by microsatellite analyses (Tables S1 and S2). Figure 2 compares the SIVcpz prevalence rates in Mitumba, Kasekela and Kalande with the corresponding population sizes over an eight-year observation period. Although fewer in number, Kalande apes consistently exhibited a much higher biannual prevalence rate (mean = 46.1±7.6%) than either Mitumba (mean = 12.7±4.9%) or Kasekela apes (mean = 12.1±3.2%) (Figure 2C). Community had a statistically significant effect on prevalence, with Kalande chimpanzees exhibiting significantly higher infection rates than Mitumba or Kasekela (Kruskal-Wallis test, P<0.001). The same relationships were also observed when annual prevalence rates were used, indicating that the statistical significance was not the result of an inflated sample size.

**Table 1:** Sample collection in Kalande.

Year	Number of fecal samples	Number of individuals sampled	Minimum population estimate	Maximum population estimate	Percent population sampled (min)	Percent population sampled (max)
2001	1	1	24	35	4.2	2.9
2002	24	11	28	36	39.3	30.6
2003	22	8	20	29	40.0	27.6
2004	69	8	15	25	53.3	32.0
2005	45	9	13	25	69.2	36.0
2006	38	9	13	22	69.2	40.9
2007	38	8	13	20	61.5	40.0
2008	29	8	14	19	57.1	42.1
2009	38	10	14	18	71.4	55.6

### Kalande Population Decline

The population sizes of the habituated Kasekela and Mitumba communities have been monitored closely since the 1980s and are based on detailed daily observational records [15], [17], [18]. Regular monitoring of the Kalande population did not begin until 1999, but intermittent population data are available from as early as 1968, due to attempts to habituate this community for observation. In 1969, C. Gale identified more than 20 individuals and estimated that the total population size of Kalande was approximately 40 individuals (unpublished data from the Gombe Stream Research Center). In 1999, E. Greengrass observed large parties of 20 or more individuals in Kalande and estimated a total population of 30 individuals (unpublished data). Between 2000 and 2002, F. Grossman saw parties of up to 16 individuals and identified a total of 30 distinct Kalande apes (unpublished data). Nest transect data for these years yielded smaller numbers, as only weaned individuals make nests, with an estimated 14.7 weaned individuals in the population in 1999–2000 [16], and 17 weaned individuals in 2000–2002 (95% CI: 12.1–24.6).

As monitoring continued, the Kalande chimpanzees became more habituated, with the median distance from observers decreasing from 43 m in 2004 to 13 m in 2009. While most of the Kalande chimpanzees remained wary, observers were generally able to determine the age-sex class of individuals, and visually identify and name at least 14 regularly seen apes (additional individuals were named only after emigrating to other communities). Despite a growing acceptance of observers, the number of chimpanzees seen at any given time decreased. During 290 observations in the time from January 2002 to June 2009, the mean party size was 2.98±1.63, and the maximum party size only nine chimpanzees. When, as is common in measures of party composition, dependent offspring were not included, the mean party size was 1.89±1.11 (range = 1 to 6). These parties were not only smaller than those observed earlier in the same community, but also much smaller than those seen in Kasekela, where the mean party size for 2002–2007 was 10.3±9.2 (dependent offspring not included; range = 1 to 38; unpublished data from the Gombe Stream Research Center).

**Figure 3:**
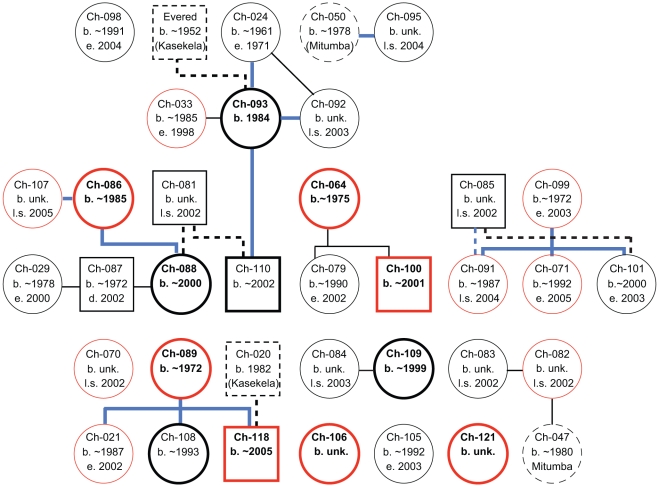
Kinship of Kalande chimpanzees. Males are shown as squares and females as circles. Individuals not known to have resided in Kalande are indicated by dashed lines. SIVcpz infected individuals are highlighted in red. Current residents of Kalande are identified by bold thick-lined shapes. Vertical lines connect parents and offspring, while horizontal lines connect siblings. Fathers are linked to offspring by dashed lines. Blue lines indicate that the relationship is significant at the P<0.001 level in KINSHIP. The diagonal connection between Ch-024 and Ch-092 indicates a likely sibling or other close matrilineal relationship.

More recently, we have estimated the Kalande population size by using sightings, genetic data, and inferences about age and residence patterns (Tables S3 and S4). Microsatellite analyses revealed maternal, paternal, and sibling relationships and thus confirmed the Kalande origin of eight Kasekela and Mitumba immigrants (Figure 3, Tables S5 and S6). Of the 26 individuals initially sampled in Kalande, three were subsequently sampled after they transferred to a habituated community, including two to Kasekela (Ch-071, Ch-099) and one to Mitumba (Ch-098). Five others were not sampled in Kalande, but were found to have either mothers (Ch-021, Ch-079, Ch-101) or other close kin (Ch-029, Ch-033) in Kalande, supporting the view that they originated there (Table S5). For three additional individuals, who immigrated to Kasekela (Ch-022, Ch-105) and Mitumba (Ch-076) in recent years (2000, 2004, and 2001, respectively), we did not find close genetic ties to Kalande, but we inferred that they came from Kalande because their origin in a habituated community could be ruled out. We have thus genetically identified 34 individuals of known (n = 26), highly likely (n = 5), or inferred (n = 3) Kalande origin.

In combination, these data have yielded minimum and maximum population estimates for Kalande. As shown in Figure 2B, the Kalande community has declined substantially. Estimates based on inferred community membership yielded a 1998 population size of 19–43 individuals. By 2002, when genetic sampling and more regular observations provided a narrower range of estimates, approximately 28–36 individuals lived in Kalande. By the beginning of 2009, only 14–18 individuals remained.

The observed decrease in Kalande party sizes corresponds to documented losses from the community through death and emigration (Tables S3 and S4). In 2002 alone, six to ten individuals died, leaving the community with only a single adult male. Moreover, the largest number of adult males observed in Kalande since 1998 (n = 4) was much lower than the number of adult males seen during intercommunity interactions in the 1970s (n = 7–9) [17], suggesting that the entire community was larger in the 1970s than it was in the late 1990s.

Of the 52 chimpanzees known or suspected to have resided in Kalande (1998–2009), only 14–18 were still living in Kalande at the beginning of 2009. Eleven had emigrated, 10–22 died, and 4 were of unknown status (Table S4). For 34 of the 52 chimpanzees, fecal samples were available for genotype and SIVcpz status determinations. Five of these sampled individuals are known to have died (Ch-021, Ch-033, Ch-085, Ch-087, Ch-099), and an additional 9 have not been sampled since 2005 (Ch-070, Ch-081, Ch-082, Ch-083, Ch-084, Ch-091, Ch-092, Ch-095, Ch-107) and are thus presumed to have died (Figure S1). Of these 14 individuals, seven (50%) were infected with SIVcpz at the time of their last sample, including three (Ch-021, Ch-033, Ch-099) of the five known dead (60%) and four (Ch-070, Ch-082, Ch-091, Ch-107) of the 9 presumed dead (44%) (Figure S1; Table S4). Three of the known dead emigrated from Kalande to Kasekela where they died of unknown causes (Ch-033), the consequences of a spinal cord injury (Ch-099) [2], and an AIDS-like illness (Ch-021) (K. Terio, personal communication). An additional female (Ch-036), who immigrated into Kasekela in 1997, possibly from Kalande, also died of an AIDS-like illness [2].

During the years in which we were able to determine SIVcpz prevalence (2002–2009), the Kalande community exhibited a negative annual growth rate, using both minimum (mean ± standard = −6.5±14.1%) and maximum (−7.4±8%) community size estimates. In contrast, the Mitumba and Kasekela communities, which had lower SIVcpz prevalence rates, both grew (Mitumba: 1.9±8.4%; Kasekela: 2.4±5.3%) (Figure 2D). The difference in annual population growth among these three communities was statistically significant, but only for the maximum estimate of the Kalande community (Kruskal-Wallis test, n = 8 years for each of 3 communities; P<0.05).

From the start of 2002 through the end of 2009, Mitumba increased from 22 to 25 individuals and Kasekela increased from 51 to 61 individuals (Figure 2A). Much of this growth, however, consisted of immigrating females and their offspring, who transferred from Kalande to Kasekela (Ch-021, Ch-071, Ch-079, Ch-099, Ch-101, Ch-105, with one infant subsequently born to each of Ch-021, Ch-071, Ch-099, and Ch-105); from Kalande to Mitumba (Ch-098); and from Mitumba to Kasekela (Ch-080, Ch-096). Some of these individuals (Ch-099 and her infant, and the infants of Ch-021 and Ch-071) died by the end of 2009. Excluding the surviving 2002–2009 immigrants and their offspring, the Mitumba community grew by one, while the Kasekela community neither grew nor declined. The net population growth in the communities with lower SIVcpz prevalence was thus largely due to female immigration from Kalande. In contrast, Kalande suffered a net population decline, even accounting for female migration, which comprised 11 of the 21–33 departures from Kalande. The remaining departures from Kalande resulted from known (10) or suspected (12) death (Figure S1, Table S4).

### Inter-Community Spread of SIVcpz

**Figure 4:**
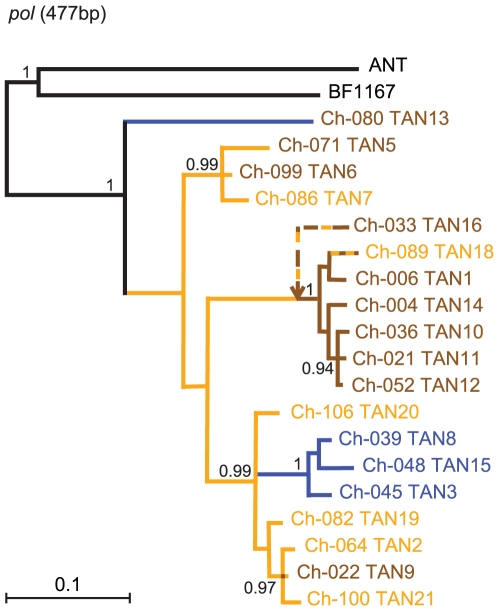
Phylogeny of SIVcpz in Gombe. A phylogenetic tree was constructed from available *pol* sequences, using SIVcpz*Pts* strains from the Democratic Republic of Congo (ANT and BF1167) as outgroups. Viruses (and their chimpanzee hosts) are color coded according to their most recent community (Mitumba, blue; Kasekela, brown; Kalande orange), while branch colors indicate the likely origin of these infections (e.g., Ch-099 resided in Kasekela from 2004 to 2006, but acquired TAN6 in Kalande). Striped lines indicate uncertain origin (genetic and demographic data suggest that Ch-033 and Ch-089 acquired their infections in Kalande and Kasekela, respectively; the location where Ch-022 acquired SIVcpz is unknown). The phylogenetic position of TAN16 is approximated (arrow) based on the position of its *env-nef* sequences (see Figure S2B in reference [2]). The tree was inferred by Bayesian methods [38]; numbers on nodes indicate posterior probabilities (only values above 0.95 are shown). The scale bar represents 0.1 substitutions per site.

To compare the evolutionary relationships of SIVcpz strains in Kalande to those in Kasekela and Mitumba, we amplified a 477-bp *pol* fragment from two chimpanzees (Ch-086, Ch-100), one of whom (Ch-086) had not previously been characterized. Attempts to amplify SIVcpz sequences from Ch-091, Ch-107, Ch-118 and Ch-121 remained unsuccessful, most likely due to sample degradation. A phylogenetic tree of SIVcpz *pol* sequences from Gombe is shown in Figure 4, with viruses color-coded according to their current or most recent community (Mitumba, blue; Kasekela, brown; Kalande, orange). Attempts to amplify a *pol* region from Ch-033 were unsuccessful and the phylogenetic position of this ape's virus (TAN16) is approximated based its gp41/*nef* region [2]. The analysis shows that all Gombe viruses form a monophyletic lineage, and that viruses from Mitumba, Kasekela and Kalande are interspersed, indicating inter-community transfers. Interestingly, all viruses, except TAN13 from Ch-080, appear to have their roots in Kalande (indicated by orange colored branches). This includes TAN5, TAN6, and TAN9, which are derived from females who are known (Ch-071, Ch-099) or inferred (Ch-022) to have immigrated from Kalande. It also includes all viruses from Mitumba, which fall within a cluster of Kalande viruses. Finally, it includes TAN14, TAN10, TAN11 and TAN12, which cluster distal to TAN16 from a female (Ch-033) inferred to have originated in Kalande. Based on these data, it seems clear that most extant viruses in Kasekela and Mitumba trace back to the Kalande community, with movement of infected females representing the main mode of inter-community transmission. Interestingly, TAN18 identified in a resident Kalande female (Ch-089) likely originated in Kasekela. Ch-089 visited Kasekela in 2003–2004 where she became pregnant, based on paternity analysis of her son (Ch-118), and presumably also acquired SIVcpz. Thus, in this case, SIVcpz was likely transmitted back to Kalande, again through the movement of a female.

The dispersal of infected females from Kalande was accelerated in 2002 when Kalande suffered an especially devastating series of deaths. Up to 10 individuals were last observed in 2002, including two adult males identified both visually and genetically (Ch-085, Ch-087), four apes known only from genotyped samples (Ch-070, Ch-081, Ch-082, Ch-083) and four known only visually (KLAM2, KLAF4, KLSM2, BB-089). Of these, Ch-087 was likely killed by people [18], Ch-085 suffered from severe diarrhea when last seen and likely died from causes unrelated to SIVcpz, and KLAF4 and KLSM2 died from respiratory disease [18]. Of six genotyped individuals, two were positive for SIVcpz when last tested (Ch-070 and Ch-082). Thus, while SIVcpz may have contributed to 2 of 6 (33%) known cases of death in 2002, the remaining mortality was likely caused by other factors. Interestingly, there was an outbreak of respiratory disease in Kalande, which appears to be linked to an outbreak first reported in Kasekela. Between September 7 and 30 of 2002, 24 of 51 Kasekela chimpanzees were seen with symptoms of respiratory illness. Subsequently, between October 5 and 15, four Kalande apes were seen coughing severely, of which two (KLAF4 and KLSM2) died, with their bodies found on October 10 and 11, respectively. Mitumba also appears to have been affected by this outbreak, with 5 of 23 Mitumba chimpanzees seen with symptoms of respiratory disease from late September to mid-October.

After these deaths, several mothers either visited (Ch-086, Ch-089, Ch-093) or permanently transferred (Ch-099) into Kasekela. Two of these females (Ch-086, Ch-099) were already SIVcpz infected, while Ch-089 apparently became infected during her visit in Kasekela. Adult females usually do not transfer once they have settled and reproduced in a community [20]. These individuals thus seem to represent extraordinary cases where the decline of their community prompted their emigration into neighboring communities. It is thus likely that the dramatic decline of Kalande chimpanzees in 2002 increased the inter-community spread of SIVcpz.

### Habitat Quality

Over the years, Kalande chimpanzees suffered a reduction of available habitat both by deforestation of land outside the park [15] and through loss of territory to the Kasekela community. Since this habitat loss and consequent reduced food availability could have contributed to the community's decline [15], we compared the abundance of chimpanzee food plants within the ranges of all three Gombe communities. We conducted a series of vegetation plots placed throughout the park, in which we determined the basal area of chimpanzee food trees (defined as the total area covered by the cross-sections of tree trunks measured at breast height [21] and counted the number of stems of smaller food plants. Because ranging data for the unhabituated Kalande chimpanzees are limited, we used two estimates of their range, a *minimal range* and a *likely range* (see Methods), which measured 597 ha and 1182 ha respectively. Because observational data for each year yielded only a limited number of locations for Kalande chimpanzees, we calculated a single 2002–2009 range using all locations. For comparison, earlier qualitative estimates for the Kalande range, based on small samples of sightings, were ∼900 ha for 1999–2000 (E. Greengrass, unpublished data) and ∼984 ha for 2000–2002 (F. Grossmann, unpublished data). The 2007 ranges for Mitumba and Kasekela covered 544 ha and 1649 ha respectively.

**Figure 5:**
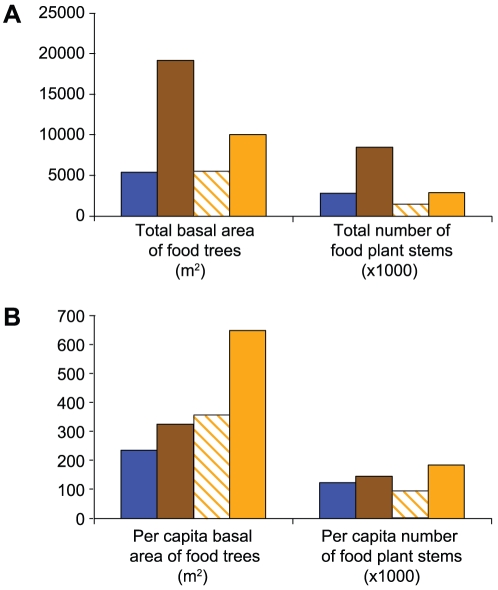
Abundance of food plants in Gombe. Data are color coded according to the range of the respective community (Mitumba: blue; Kasekela: brown; Kalande minimal range: vertical orange hatching; Kalande likely range: solid orange). (A) Total basal area of food trees (m^2^) and total number of stems of smaller food plants (vines and shrubs, shown in thousands of stems). (B) Per capita abundance of food plants. Population sizes are from 2007, using the median estimate for Kalande, and food plant abundance data are from 2007–2009. Range data for Kasekela and Mitumba communities are from 2007. Because available range data are more limited for Kalande, we used all available information from 2002–2009.

We found that the estimated total basal area of chimpanzee food trees in Kalande (5,420–10,050 m^2^) was intermediate between that of Mitumba (5,403 m^2^) and Kasekela (19,155 m^2^) (Figure 5A). However, because few chimpanzees lived in Kalande, the per capita abundance of food trees was actually highest in Kalande (Mitumba: 235 m^2^; Kasekela: 325 m^2^; Kalande: 356–648 m^2^) (Figure 5B). In addition to fruit and leaves from trees, chimpanzees obtain much of their food from smaller plants, including vines and shrubs. The range of estimates for the abundance of these plants in Kalande (1,432,000–2,841,000 stems) encompassed the estimate for Mitumba (2,820,000 stems), but was much lower than in Kasekela (8,485,000 stems) (Figure 5A). However, as with the food trees, the small population in Kalande resulted in relatively high per capita estimates of smaller plants (92,000–183,000 stems), with the upper end of this range exceeding the estimates for both Mitumba (123,000 stems) and Kasekela (144,000 stems) (Figure 5B). Thus, while the density of food species was lower, the large size of the Kalande range resulted in an overall per capita abundance of food species that was comparable to, or larger than, those of the other Gombe communities. The predicted population size for this range, assuming levels of per capita food abundance comparable to those in Mitumba, was 24–43 individuals based on food trees, and 12–23 individuals based on the abundance of other food plants. The estimated population of the Kalande community in 2002 (32–37 individuals) was thus within the range of the population predicted from the abundance of food trees, but larger than the population based on the abundance of other plants.

### Modeling the Impact of SIVcpz Infection on Chimpanzee Population Growth

To estimate the impact of SIVcpz infection on chimpanzee population growth, we employed two sets of models: deterministic calculations of critical prevalence and event-based stochastic simulations. Deterministic calculations tend to provide an upper bound for population growth, since stochastic factors generally reduce population growth. However, stochastic simulations incorporate random processes likely to occur in real populations, particularly the small populations typical of chimpanzee communities.

**Figure 6:**
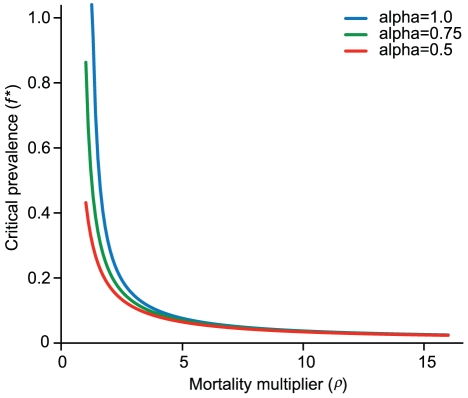
Predicted critical prevalence of SIVcpz (f^*^) for different levels of the mortality multiplier (*ρ*). Three different lines are given for different levels of the fertility multiplier (*α*). The most recent empirical estimates for these parameters are *ρ* = 10–16 and *α* = 0.5.

The deterministic model indicated that the *critical* prevalence of SIVcpz (i.e., the prevalence below which the population does not decline) depends greatly on the mortality multiplier, *ρ*, which reflects the excess mortality due to SIVcpz infection (Figure 6). We previously found that SIVcpz infection increases the mortality hazard 10–16 fold, depending on assumptions concerning the death and infection status of certain individuals [2]. Because the 95% confidence intervals for these estimates were wide (e.g., 2.8–34.3 for the lower estimate [2]), we considered a range of estimates for *ρ*. When SIVcpz doubles mortality (*ρ* = 2), the prevalence can be 17% and the population will not decline. When *ρ* = 5, the prevalence can only be 6.4%, and when *ρ* = 10, the maximum prevalence compatible with population persistence is 3.4%. Thus, if the actual value for *ρ* is indeed in the range of 10–16, even relatively low prevalence rates of SIVcpz are predicted to lead to population decline.

SIVcpz infection may affect population growth not only by increasing mortality, but also by reducing fertility. Infected females studied to date experienced a reduction in fertility, α, of 50% [2]. Because this estimate is based on a small sample size, we examined the impact of three different values of *α* on the critical prevalence (Figure 6). We found that the effect of SIVcpz infection on fertility affects the critical prevalence only if the mortality multiplier is low (Figure 6).

The critical prevalence determination rests on the assumption of a large population size. It is well known in conservation ecology that small population size, and the stochastic variation introduced by demographic “sampling” from such populations, can have a powerful effect on population outcomes [22]. Compared to the assumptions of the deterministic model, the typical chimpanzee community is “small,” especially when considered in isolation from other communities. To investigate the effects of SIVcpz infection in such populations, we developed stochastic simulations that incorporated both demography and infection dynamics.

For the stochastic simulations, we used our best estimates of epidemiological and demographic parameters. We used two methods for estimating *τ*, the probability of transmission of SIVcpz per coital act: (i) the transmission probability for HIV-1 estimated by Gray and colleagues using sero-discordant human partnerships in Rakai, Uganda [23], and (ii) the transmission probability for SIVcpz based on the following parameters calculated from data from the Kasekela chimpanzees: the basic reproduction number, *R_0_*; the average copulation rate for each sex; the median number of susceptible males and females; the baseline mortality rate for each sex; and the SIVcpz associated mortality multiplier, *ρ* (see Methods for details). As for the deterministic model, we used a range of values for *ρ*, calculating *τ* based on both high (*ρ* = 10) and low (*ρ* = 5) estimates. The resulting values for SIVcpz (*τ* = 0.00077–0.0015) bracket the value for HIV-1 (*τ* = 0.0011). In addition, calculations from annual incidence/prevalence ratios observed in the Kasekela community among sexually active individuals yielded estimates consistent with these values.

Simulations started with a population based on data from the Kasekela community during years (2002–2007) for which we have demographic, epidemiological and mating data. We chose Kasekela as a model community both because this is the community for which we have the most detailed data, and because Kasekela's size in these years (median = 55.5 individuals, range = 51–61) is close to the average size of chimpanzee communities in other long-term studies (median = 47.1, range = 10–144, n = 8 [24]). We ran simulations for 12 sets of starting conditions, with varying combinations of the following parameters: migration both in and out of the community (allowed or not allowed); the percentage of incoming females infected with SIVcpz (*p_F_* = 5% or 30%), transmissibility (*τ* = 0.00077, 0.0011, or 0.0015); and the mortality multiplier (*ρ* = 5 or 10). For each set of starting conditions, we ran 10,000 replicate simulations over a 100-year time frame, and calculated the percentage of chimpanzee populations that persisted 100 years, the percentage of those surviving populations in which the virus persisted, the population growth rate of the surviving chimpanzee populations, and the mean time of extinction for those chimpanzee populations that did not persist (Table 2).

**Table 2:** Stochastic modeling of chimpanzee population growth.

	Transmission probability (*τ*)^1^		Mortality multiplier (*ρ*)	SIVcpz prevalence in migrating females (*p_F_*)^2^	Population persistence (%)^3^	SIVcpz persistence (%)^4^	Population growth rate^5^	Mean extinction time (years)^6^
No migration	SIVcpz	0.0015	10	–	71	18	−0.018	49
		0.0008	5	–	58	37	−0.019	50
	HIV-1	0.0011	10	–	65	29	−0.019	56
		0.0011	5	–	40	59	−0.020	43
Migration	SIVcpz	0.0015	10	0.05	99	2	0.033	24
		0.0015	10	0.30	99	2	0.021	27
		0.0008	5	0.05	87	21	0.025	39
		0.0008	5	0.30	88	15	0.015	40
	HIV-1	0.0011	10	0.05	98	4	0.031	30
		0.0011	10	0.30	97	5	0.019	38
		0.0011	5	0.05	66	39	0.021	48
		0.0011	5	0.30	66	30	0.011	52

1 SIVcpz refers to transmission probabilities of SIVcpz estimated from empirical epidemiological and demographic data in Gombe. The empirically-derived values of *τ* = 0.000769 and *τ* = 0.001539 were calculated for values of *ρ* = 5 and *ρ* = 10 respectively (see Methods for details). HIV-1 refers to the transmission probability of HIV-1 as estimated by Gray and colleagues [23] for sero-discordant couples in Rakai, Uganda.

2 Probability that a migrating female is SIVcpz infected; low and high *p_F_* values reflect the range of prevalence rates determined for wild chimpanzee communities [11], [55].

3 Percent of chimpanzee populations that survive 100 years of simulation.

4 Percent of chimpanzee populations that remain SIVcpz infected after 100 years of simulation.

5 Growth rate for chimpanzee populations surviving 100 years of simulation.

6 Mean time to extinction for chimpanzee populations that do not survive 100 years of simulation.

**Figure 7:**
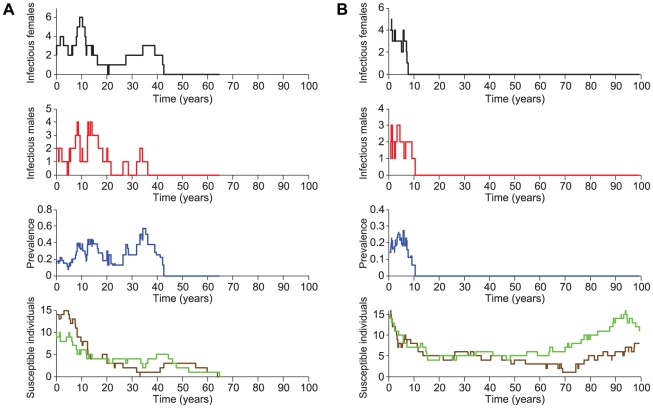
Stochastic simulations. The number of infectious females, the number of infectious males, the SIVcpz prevalence, and the number of susceptible individuals (females in green, males in brown) are shown (y-axis) in relation to time in years (x-axis). (A) Representative run illustrating population extinction. (B) Representative run illustrating SIVcpz extinction.

Overall, within the range of our starting conditions, chimpanzee populations usually persisted, whereas the virus frequently became extinct. Figure 7 depicts the results of two example runs of the stochastic simulation, showing the number of infectious males and females, the SIVcpz prevalence, as well as the number of susceptible individuals over time. In Figure 7A, the virus persisted for over 40 years, driving the chimpanzee population to extinction by year 65, while in Figure 7B the virus went extinct by year 10 and the chimpanzee population persisted.

For both chimpanzees and virus, the particular pattern of persistence varied greatly depending on the values chosen for the other parameters, as did the growth rate of surviving chimpanzee populations. In particular, migration had a profound effect on the qualitative results. Simulations with migration resulted in high persistence of the chimpanzee population (median = 93%, range = 66–99%), low persistence of the virus (median = 10%, range = 2–39%), and robust population growth rates (median = 0.021, range = 0.011–0.033; Table 2). In contrast, simulations without migration resulted in lower persistence of chimpanzee population (median = 62%, range = 40–71%), and greater persistence of the virus (median = 33%, range = 18–59%), and those chimpanzee populations that did not go extinct suffered negative population growth rates (median = −0.019, range = −0.020–−0.018; Table 2). This latter point indicates that while the populations did not go extinct within the time bounds of the simulation, they were almost certain to go extinct shortly thereafter. For chimpanzee populations that went extinct within the 100-year time frame, the time to extinction was fairly uniformly distributed across this time span. While migration reduced the overall probability of extinction, those populations that did go extinct tended to do so sooner in simulations with migration (median = 39 years, range = 23–52) than without migration (median = 50 years, range = 43–56). Whether incoming females had a low or high probability of being infected with SIVcpz had a negligible effect on chimpanzee population persistence (median persistence = 93% for *p_F_* = 5% and 30%) and viral persistence (*p_F_* = 5%, median persistence = 13% vs. *p_F_* = 30%, median persistence = 10%), but did result in substantially slower population growth for surviving chimpanzee populations (*p_F_* = 5%, median growth = 0.028 vs. *p_F_* = 30%, median growth = 0.017).

The effect of the SIV-excess mortality multiplier (*ρ*) had a modest and perhaps counter-intuitive effect on both chimpanzee and viral persistence. For example, in simulations that included migration, chimpanzee population persistence was higher when SIVcpz-induced mortality was high (*ρ* = 10, median persistence = 99% vs. *ρ* = 5, median persistence = 77%). In contrast, viral persistence was lower when SIVcpz-induced mortality was high (*ρ* = 10, median persistence = 3.1% vs. *ρ* = 5, median persistence = 26%). These patterns are interrelated, because higher mortality causes infected individuals to exit the population more quickly. In a small population, even a small number of such exits can lead to viral extinction. With lower disease-induced mortality, the sojourn time of infectious individuals is longer, leading to more potential infection.

## Discussion

In this study, we examined the impact of SIVcpz infection on chimpanzee population growth in three Gombe communities. We found that a high prevalence of SIVcpz was associated with population decline: the Kalande community, which exhibited a SIVcpz prevalence of ∼40% to 50% for at least a decade, suffered a population decline during this same observation period. In contrast, the Mitumba and Kasekela communities, which exhibited lower prevalence rates, experienced mean population growth of 1.9% and 2.4%, respectively. These growth rates were influenced by the movement of females among the various communities. Controlling for female migration, Kalande still declined, Mitumba grew by one individual, and Kasekela remained stable. Consistent with this, our deterministic demographic calculations predict that even modest levels of SIVcpz prevalence can lead to population decline.

The decline of the Kalande community resulted from both death (10–22 individuals) and emigration (11 individuals). Of 34 individuals whose infection status was known, 5 are known to have died, and an additional 9 have not been sampled since 2005 and are thus presumed to have died (Figure S1). Of these 14 individuals, seven (50%) were infected with SIVcpz at the time of their last sample. Although the extent to which SIVcpz contributed directly to the mortality in Kalande remains unclear in the absence of necropsy data, any virus induced increase in mortality is likely to have amplified the factors promoting emigration from Kalande. Emigration of adolescent females is typical of chimpanzees [25] and by itself is unlikely to lead to population decline, as it is usually matched by immigration from other communities. However, emigration in Kalande was exacerbated by two factors: emigration of mothers with dependent offspring, and lack of immigration from other communities. Mothers with dependent offspring rarely emigrate, presumably due to risk of infanticide by males in the new community [26], [27]. “Mass transfer” of females with dependent offspring occurred in Mahale, when the K-group declined to a single adult male [28]. A similar process occurred in Kalande. The death of adult males in 2002 likely prompted females, including mothers with dependent offspring, to search for a community with more males, and thus more mates and improved protection. While only one mother (Ch-099) and her offspring (Ch-071, Ch-101) departed Kalande permanently, several other mothers are known (Ch-086) or suspected (Ch-089, Ch-093) to have visited Kasekela, and Ch-089 apparently visited long enough to conceive an infant and acquire SIVcpz infection. Moreover, immigration has failed to replace the emigrants. A decline in adult males since the 1980s is likely to have led to a decreased ability to defend territory, a decreased home range size, and a decreased ability to attract new females. Whether SIVcpz contributed directly to the deaths of some of the Kalande males that appeared to have precipitated the subsequent wave of female emigration is not known. One of the males, Ch-087, who was killed by people, was negative for SIVcpz. A second male, Ch-085, who died of disease, was also negative for SIVcpz. The infection status of the remaining two males, KLAM2 and KLSM2 (Table S3), could not be determined because neither appears to have been sampled. It is possible that one of them was Ch-081, who was SIVcpz negative when last sampled. However, it is equally possible that Ch-081 represents another adult male, such as KLAM4, who was seen as recently as 2010 and also has not yet been sampled. Thus, while two of the four males who died in 2002 were SIVcpz negative, we do not know the infection status of the other two. Given the high infection rates in Kalande, it is thus possible that SIVcpz contributed to their death.

In addition to SIVcpz, chimpanzees in Gombe face other threats. In particular, poaching and loss of habitat outside the park represent significant hazards of survival, particularly for the Mitumba and Kalande communities, as these communities border dense human settlements [15], [18]. Individuals from both communities are suspected to have been killed by people [15], although conclusive evidence exists for only one of these cases [15]. Habitat loss occurred through the conversion of forest and woodland habitats adjacent to the park to human croplands and settlements [15]. Estimates of the overall abundance of trees, shrubs and vines that provide food for chimpanzees, however, indicate that the current Kalande population is low compared to its potential food supply (Figure 5). The Kalande chimpanzees live in the southern half of Gombe, which compared to the rest of the park has less evergreen forest and more open woodland and grassland [15] (Figure 1). These more open habitats provide a lower density of food plants, especially vines and shrubs. Nonetheless, the likely range of Kalande (1182 ha) is twice that of Mitumba (544 ha), and thus contains a total food supply that exceeds that of Mitumba. It therefore seems likely that the Kalande range could support at least as many chimpanzees as the Mitumba range (n = 25), rather than the much smaller number of apes currently found in Kalande (n = 14–18). Data on habitat quality for years preceding 2007 are not available. Thus, habitat loss may have contributed to the early decline of the Kalande community. However, the current abundance of food plants within the Kalande range suggests that the more recent decline of this community's population cannot be explained by habitat loss alone. This view is further supported by C. Gale's observations from 1968–69, when the Kalande community numbered at least 40 individuals, frequently foraged in large parties, and apparently found ample food within the same valleys used by the community in more recent years (unpublished data from the Gombe Stream Research Centre).

Phylogenetic analysis of SIVcpz sequences revealed the likely direction of viral spread both within and between communities. All but one of the major viral lineages in Gombe are plausibly rooted in Kalande, suggesting a key role for Kalande in the spread of SIVcpz infection. The increase in emigration and visiting of neighboring communities by females that followed the death of four Kalande males in 2002 thus likely increased the spread of the virus throughout Gombe.

In contrast to Kalande, the Mitumba and Kasekela communities did not decline during the study period, despite having infection rates well above the predicted critical prevalence. The persistence of these communities could be interpreted to mean that we have assumed an overly high estimate of *ρ*, the increase in mortality caused by SIVcpz infection. However, recent findings render this explanation unlikely. Since our initial report [2], the Kasekela community has experienced three additional SIVcpz-related deaths (Ch-021 and her infant, and Ch-033), while none of the uninfected individuals died or disappeared. Moreover, one of the infected chimpanzees (Ch-021) whose body was recovered suffered from CD4+ T cell depletion and immune system destruction (K. Terio, personal communication). Finally, because chimpanzees mature and reproduce slowly, even a very low value for *ρ* is predicted to lead to a population decline at the observed prevalences. Instead, we believe that two other factors are responsible for the absence of a population decline in Mitumba and Kasekela. First, much of the observed growth in these communities depended on immigrants and their offspring. Excluding the surviving 2002–2009 immigrants and their offspring, the Mitumba community grew by one, while the Kasekela community neither grew nor declined in numbers. Second, the current infections in Mitumba and Kasekela are largely the result of very recent transmission events. In humans, the median time from HIV infection to death in untreated patients in rural Uganda without access to anti-retroviral therapy was 9 years [29]. The current infections may therefore take several more years before they have a measurable impact on population growth.

Deterministic calculations make the assumption of a large population size, which does not apply to most chimpanzee communities. To investigate the effects of SIVcpz infection on more representative populations, we developed event-driven stochastic simulations that incorporated both demography and infection dynamics. For these analyses, we first estimated the probability of SIVcpz transmission per coital act for Gombe chimpanzees using mortality, mating and SIVcpz prevalence data from the best-studied Kasekela community. Using conservative values of excess mortality, we estimated the corresponding SIVcpz transmission probabilities to be 0.0015 (*ρ* = 5) and 0.0008 (*ρ* = 10). Interestingly, these values bracket the transmission probability of HIV-1 reported for discordant human couples (0.0011) [23], and thus suggest that the biology of HIV-1 and SIVcpz heterosexual transmission is fundamentally similar. We also incorporated intercommunity migration into the stochastic model, including both immigration and dispersal of females. The stochastic modeling revealed that under most simulated conditions a large proportion of SIVcpz infected communities survived. This was the case both in the presence and absence of migration, although in the presence of migration the fraction of persisting communities was considerably larger (Table 2). Using a transmission probability of 0.0015, a mortality multiplier of 10, and a 30% infection probability in immigrating females, we found that in the presence of migration 99% of simulated populations persisted, with only 2% still harboring SIVcpz. In the absence of migration, only 71% of simulated populations persisted, with 18% still harboring SIVcpz (Table 2). Moreover, under the conditions tested, we uniformly found that simulations that included migration had positive mean growth rates for surviving populations, whereas in the absence of migration, even those populations that survived 100 years had negative mean growth rates and were thus at risk of going extinct. These data thus indicate that intercommunity migration protects against SIVcpz-driven extinction. Interestingly, whether incoming females had a low or high probability of being infected with SIVcpz had a negligible effect on both chimpanzee population and viral persistence. This is because the product of the probability of a given female migrating and the probability of that female being infected (assuming they are independent events) is small, regardless whether the assumed infection rates are high or low. Allowing migration led to a substantially higher rate of chimpanzee population persistence and a lower rate of viral persistence. These two outcomes are clearly related. A population in which all infections have died out but which loses all breeding females, can be rescued from extinction (at least immediate extinction) by an uninfected immigrant. This protective effect likely results from the fact that even with a 30% probability of immigrating females being infected, the majority of immigrating females are uninfected. Migration also allows infected females to emigrate, providing an additional mechanism (beyond death) for reducing prevalence.

We also explored the impact of transmission probabilities (*τ*) that were higher than those empirically determined. For example, when τ was three times higher than our highest estimated value, less than 10% of the simulated chimpanzee populations persisted for the duration of simulation (not shown). Overall, higher values of *τ* revealed three consistent trends: (i) chimpanzee population persistence decreased, (ii) viral persistence increased, (iii) the effect of the mortality multiplier (*ρ*) on chimpanzee/viral persistence became attenuated. While such transmission probabilities are perhaps implausibly high, there are conditions such as acute-to-acute infection or genital ulcer disease that are known to greatly increase the transmission probability of HIV-1 [30]. Indeed, the sudden appearance of new infections in Mitumba (2003) and Kasekela (2005) suggested a series of acute-to-acute transmissions in both communities [2]. Thus, increased transmissibility of SIVcpz would be expected to have a negative impact on the survival of infected communities.

Our mathematical models thus lead to three major conclusions. First, SIVcpz infection can cause serious population decline. Second, depending on the population structure and transmission dynamics, SIVcpz may be more likely to go extinct than its chimpanzee host. In fact, most infections are predicted by our models to die out under current epidemiological conditions. Third, intercommunity migration is a key parameter that increases survival of infected populations, even when the immigrating females have a chance of being infected with SIVcpz.

In conclusion, we found that a chimpanzee community in which roughly half of all members harbored SIVcpz suffered a dramatic population decline, and that even moderate levels of SIVcpz infection are predicted to result in negative population growth. At the same time, simulations indicated that in representative populations, the virus frequently died out before the host population, especially when intercommunity migration was incorporated into the model. Thus, population extinction is not an inevitable consequence of SIVcpz infection. Instead, the fate of an infected community depends on the particular epidemiological and ecological circumstances that are unique to that community. These results are consistent with the uneven distribution of SIVcpz infection in wild chimpanzee populations: infected communities either become extinct, after which the vacated habitat may be colonized by uninfected chimpanzees, or the virus goes extinct, allowing the previously infected population to recover.

The finding that SIVcpz is pathogenic in chimpanzees reveals yet another challenge to chimpanzee conservation. This challenge is sobering given that few options exist for intervention. For a number of reasons, including the need for daily drug administration, treating wild chimpanzees with antiretroviral therapy is currently not feasible. Although the persistence of endemically infected chimpanzee populations across central Africa [10], [11] would argue that this threat is not catastrophic, the long-term consequences of SIVcpz infection in the context of other survival hazards remains to be determined. Also, while little can be done to control the virus spread within a population, it is clear that the risk of introducing SIVcpz infection into a naïve population has to be taken into account in conservation planning. For example, it is critically important that efforts to reintroduce chimpanzees to the wild include SIVcpz screening to prevent the release of infected individuals. Additionally, the risk of exposing uninfected communities to SIVcpz should also be taken into account in the cost-benefit analysis when planning corridors between isolated chimpanzee populations.

## Materials and Methods

### Study Site

Gombe National Park is located in northwestern Tanzania, along the eastern shore of Lake Tanganyika. The park's southern border is located 15 km north of Kigoma. The park covers 35 km^2^ of rugged terrain, rising from the lakeshore in the west (770 meters above sea level; m.a.s.l.) to the crest of the rift escarpment in the east (1300 to 1600 m.a.s.l.) [15], [17], [18]. As of January 2009, the park provides habitat for 96–100 chimpanzees in three communities: Mitumba (25), Kasekela (57), and Kalande (14–18). Most research has focused on the Kasekela community, which Goodall began studying in 1960 [17]. Efforts to habituate the Mitumba community began in the 1980s and by the mid-1990s most Mitumba chimpanzees could be observed within a distance of 20–30 meters [18]. Efforts to habituate the Kalande community started with a six-month project by C. Gale (December 1968–June 1969), followed by additional attempts in the 1970s and 1980s, which were not successful. However, a monitoring program initiated by E. Greengrass (February 1999–August 2000) and F. Grossmann (September 2000–March 2002) has continued to the present. In this program, researchers have not attempted to habituate chimpanzees, but have instead focused on nest transect surveys (1999–2002), monitoring of phenology trails (2002 - present), and opportunistic sightings of chimpanzees and other wildlife (1999 - present). Since 2002, Tanzanian field assistants trained by Greengrass and Grossmann have continued the monitoring, conducting regular searches of the area for chimpanzees and other wildlife.

### Sample Collection

Fecal and urine sample collection in Gombe began in 2000, with collection of feces starting in Kalande in late 2001. For habituated apes, fecal and urine samples were collected under direct observation [2], [10]; however, this was not possible for most Kalande apes, who were sampled by collecting stool from the forest floor near night nests. When possible, field assistants also collected samples during direct observation, but because of the brief observation times at Kalande, only few such opportunities occurred. Fecal samples (∼20 g) were placed into 50 ml conical tubes, and mixed with equal amounts of RNA*later* (Ambion). If the sample was collected under direct observation, the name (if known) or age-sex class was recorded. Time, date, location, and name of collector were also recorded. Specimens from Kasekela were frozen on the day of collection, while specimens from Mitumba and Kalande remained at ambient temperature until transported to the field lab in Kasekela (usually within one week of collection). Samples were shipped at ambient temperatures, then stored at −80°C upon receipt. Between 2000 and 2009, a total of 1,536 fecal samples were collected from all three Gombe communities, 1,153 of which have been reported previously [2]. During the same time period, 341 fecal samples were collected from 26 individuals who resided in Kalande (Table S1). Three Kalande apes transferred to Kasekela or Mitumba during the study years and were previously reported (Ch-071, Ch-098, Ch-099). A fourth female, Ch-108, was sampled in Kasekela, but was apparently visiting rather than transferring, as she has since been sampled in Kalande. All individuals were identified by microsatellite genotyping. A median 4.5 samples were collected for each Kalande chimpanzee (range = 1–75). Table 1 summarizes the number of samples collected in Kalande for each year since 2001.

### Sample Identification and Genotyping

Fecal DNA was extracted as described previously [2], [10], [11] and quantified using real-time PCR [31]. All individuals for whom fecal DNA was available were microsatellite genotyped at autosomal loci as well as typed for sex and mitochondrial haplotype [32], [33]. A total of 116 individuals from the three communities were genotyped at a minimum of 8 of 11 microsatellite loci and were tested for relatedness.

### Identifying Parents

We used the likelihood-based program CERVUS 2.0 [34] to identify parent-offspring relationships (Tables S5 and S6). We first examined individuals within the same mitochondrial haplotype for mother-offspring relationships since mitochondrial DNA is matrilineally inherited. Females were only considered candidate mothers if they shared at least one microsatellite allele at each locus. Simulations were run using 100,000 cycles, 1% error rate, and confidence levels of 80% and 95%. The sampling proportions for the simulations were determined by including all genotyped females of a given haplotype with an additional 50% unsampled female candidates included to account for any ungenotyped females from the Kalande community. When a probable mother-offspring relationship was identified, we used the possible mother as the “known parent” in CERVUS to identify potential fathers amongst all sampled males (n = 49) from the three communities using the same simulation conditions. A male was only considered a probable father if, given the genotype of the corresponding mother, he did not have microsatellite allelic mismatches with the genotype of the presumed offspring (Table S6). In some cases, CERVUS assigned a particular candidate as “most likely,” even though a statistically significant parent was not identified.

### Kinship

To further validate parent-offspring relationships and identify siblings we also used the microsatellite genotypes to perform KINSHIP analyses [35] (Tables S5 and S6). These analyses tested whether dyads were maternally or paternally related compared to the null hypothesis that they were unrelated. We used KINSHIP to calculate a likelihood ratio for the primary (related) and null hypotheses for each dyad. Given the availability of long-term demographic data in Gombe, we were able to include the identity of known mothers and fathers for numerous individuals within the population, which improved the likelihood calculations. Nonetheless, when individuals did not have identified parents, KINSHIP was unable to differentiate between maternal and paternal lineages among autosomal loci. We also used KINSHIP to estimate the relatedness of individuals, *R*, defined as the probability that the same allele found in two individuals is identical by descent, taking into account the frequency of the allele in the population [35]. For diploid, sexually reproducing species, *R* should be 0.5 for parent-offspring and full-sibling relationships, and 0.25 for half-sibling and grandparent-grandoffspring relationships. Departures from these expected values may occur when calculating *R* from a relatively small number of loci, such as the 8 to 11 loci that were used here (which were nonetheless sufficient to correctly assign close relationships, e.g., parent, half-sibling; [36]). Thus, we obtained calculated estimates for *R* that were close to (but not precisely equal to) 0.5 for parental relationships (mother-offspring: n = 12, median = 0.43, range = 0.21–0.62; father-offspring: n = 7, median = 0.40, range = 0.24–0.74) and close to zero for unrelated individuals (n = 17, median = 0.06, range = −0.22–0.38). Tables S5 and S6 summarize all CERVUS and KINSHIP results, with particular focus on SIVcpz-infected chimpanzees from Kalande. These results are conservative in that we only report results for dyads that are (i) within the same mitochondrial haplotype and also lack microsatellite allelic mismatches; and/or (ii) significant relationships from CERVUS and the corresponding KINSHIP analyses for these dyads; as well as (iii) results for any dyad for which KINSHIP found a strongly significant relationship (P<0.001). Finally, we included results for dyads suspected to be related based on other significant dyadic relationships (i.e., if individuals A and B were related and individuals B and C were related, then we also reported results for individuals A and C).

### SIVcpz Antibody Detection

All fecal samples were screened for the presence of SIVcpz specific antibodies by enhanced chemiluminescent Western blot analysis [2], [9], [10]. Sample integrity was confirmed using an IgG control.

### Viral RNA Extraction and Amplification

SIVcpz sequences were amplified from Kalande apes Ch-100 and Ch-086 as previously described [2], [9], [10]. Briefly, fecal RNA was extracted using the RNAqueous Midi-kit (Ambion). Reverse transcription polymerase chain reaction (RT-PCR) amplification was the performed using the following primers: PTS-midpol-F1 (5′-CWAAYCAACAAGCAGARYTATGGGC-3′), CPZ-pol-R1(5′-ACBACYGCNCCTTCHCCTTC-3′), PTS-midpolF2 (5′CAAAGTGACTCYCCCATAGTAGAG-3′), and PTS-midpol-R2(5′-CCCAATCCCCCCTTTTCTTTTAAAATT-3′). RT-PCR products were gel purified and sequenced directly. The newly derived SIVcpz sequences are available at GenBank under accession numbers GU992204 (TAN7) and GU992204 (TAN21).

### Phylogenetic Analysis

To determine the evolutionary relationships of the Gombe viruses to each other and to SIVcpz*Pts* reference strains, a phylogenetic tree was constructed from available *pol* nucleotide sequences (477 bp). These included previously reported sequences from Gombe (TAN1, GenBank accession number AF447763; TAN2, DQ374657; TAN3, DQ374658; TAN5, FJ895394; TAN6, FJ895395; TAN8, FJ895403; TAN9, FJ895405; TAN10, FJ895398; TAN11, FJ895399; TAN12, FJ895400; TAN13, FJ895393; TAN14, FJ895397; TAN15, FJ895404; TAN18, FJ895396; TAN19, FJ895402; and TAN20, FJ895401), newly derived sequences from Gombe (TAN7, GU992204; TAN21, GU992204) and two SIVcpz*Pts* strains from the Democratic Republic of Congo that served as an outgroup (ANT, U42720; BF1167, FJ869116). Nucleotide sequences were aligned using CLUSTAL W [37]; sites that could not be aligned unambiguously were excluded. Trees were inferred by Bayesian methods [38].

### SIVcpz Prevalence

SIVcpz prevalence rates were determined for Kasekela, Mitumba, and Kalande apes separately from 2002 to 2009. For this analysis, individuals were considered SIVcpz positive if they had detectable antibodies in their urine or feces as determined by Western blot analysis [2], [10]. A positive Western blot is diagnostic of SIVcpz infection, except for nursing infants who may contain maternal antibodies in their feces [2]. Prevalence rates were calculated semiannually by dividing the number of positive individuals by the total number of apes tested in each community. Since SIVcpz infection is a chronic, life-long infection, we could infer the infection status for a number of missing time points, using the following guidelines: (i) if an individual was infected or uninfected before and after a missing time point, we inferred the same status for the missing time point; (ii) if an individual died after testing positive for SIVcpz, we assumed the individual was infected for all time points between the positive sample and death; (iii) if data from missing years could not be inferred according to these guidelines, the individual was omitted from prevalence calculations for that time period.

### Population Estimates

Annual population estimates for the Mitumba and Kasekela communities were based on detailed demographic records of known individuals, combined with genotyping to track individuals moving between communities. We used two methods to estimate the number of individuals in the Kalande community: (i) nest transects and (ii) a table of annual membership, based on visual identification of individuals and genetic markers.

#### Nest transects

Initial surveys were conducted by E. Greengrass (February 1999–August 2000) and F. Grossman (September 2000–February 2002). Greengrass used the marked nest count method [39], repeatedly walking transects and marking all new nests observed. This was done within the appropriate time frame, to ensure that all new nests were counted and did not decompose before they were observed. Greengrass conducted transects along three paths placed at regular intervals within the Kalande community's range. Each transect started at the lakeshore and continued in an east-northeast direction to the top of the rift escarpment. They ranged from two to four km in length. Each transect was walked every one to two months. To determine the visible transect area, Greengrass placed markers at regular intervals, indicating the maximum perpendicular distance that could be clearly observed on either side of the transect. This was done in wet and dry seasons due to potential changes in vegetation cover, although in practice seasonal changes had little impact on visibility for these transects. These data were used to calculate the nest density, defined as the total number of new nests built per day in the area covered by the transects. The nest density was then multiplied by the area of the Kalande range (estimated to be 9 km^2^) to give the total number of nests built per day.

Grossman established two transect lines located within the Kalande range, covering 11.04 and 10.30 km. Transect lines were placed as cross-country loops that avoided existing trails, traversed the area's major vegetation types and included approximately equal distances of high and low elevation. Grossmann and one field assistant walked each transect in about seven to eight hours seven times during the study period (October 2001–March 2002), yielding a total distance sampled of 149.38 km. Distances along each transect were measured with a Hip Chain distance measurer. Transect paths and nest locations were mapped with a Garmin 12XL GPS. Whenever chimpanzee nests were encountered, the perpendicular distance from the transect line to the nest was determined. Line transect data were analyzed using DISTANCE software [40], with the average rate of daily nest production assumed to be 1.15 nests per day per individual [41]. Nest disappearance was estimated at 113.6 days, based on field observations. In DISTANCE, animal density estimates are inversely proportional to the decay rate of the animal sign (in this case, nests). Grossmann (unpublished data) did not report a variance for the estimate of decay, but previous studies have reported decay rates with coefficients of variance of 7.8–13.3% [42].

#### Annual membership in Kalande

To infer the annual Kalande membership, we used (i) reports of dead chimpanzees found within the Kalande range and neighboring village land; (ii) observations from intercommunity encounters, including lethal attacks [43]; (iii) descriptions of individuals from visual observations; and (iv) identification of unique individuals from genotyped fecal samples; these data are summarized in Tables S3 and S4. Age-sex class, migration history, and relationships to other chimpanzees in the population were further clarified by matching genetic identification to notes from direct observations and pairwise tests of kin relatedness (Tables S5 and S6). Individuals who immigrated into habituated communities, and for whom close relatives were found in Kalande, were inferred to have originated in Kalande. Immigrants without close relatives in Kalande were included among the suspected former members of Kalande, although some or all of these individuals may have immigrated from relict populations outside the park. Each individual inferred or suspected to have resided in Kalande received a row in the annual membership table (Table S3). Columns for each year kept track of whether that individual was inferred or suspected to have been present in Kalande at the beginning of the calendar year. For each year, we calculated two estimates of population size: a minimum estimate, based on the individuals known or inferred to have been present, and a maximum estimate, which also included those individuals that were suspected to be present. Whenever possible, we estimated the age of each observed individual based on comparison with chimpanzees of known age. For individuals known only from genotype, we made the following assumptions: (i) they were present for three years prior to their first sample (because samples are rarely collected from infants under three years old); (ii) they were possibly present prior to that (because, if they were adults, they may have been living in the community for many years); (iii) they were possibly present for three years after their last sample (because sampling of this unhabituated community was not comprehensive every year); (iv) if an individual was not sampled for four consecutive years, we assumed death or emigration (because even rarely seen individuals are likely to be sampled at least once in three years if still alive).

### Habitat

Habitat quality was estimated using 91 20×20 m vegetation plots distributed across the park (30 plots each in Mitumba and Kalande, and 31 in Kasekela), following methods described previously [44]. Plots were stratified according to vegetation class (evergreen forest/vine tangle, thicket woodland, open woodland, grassland), which was determined by remote sensing and confirmed by on-the-ground classification. Within each plot, the Diameter at Breast Height (DBH, with breast height = 137 cm) for each tree with DBH≥10 cm was measured and used to calculate the tree's basal area. Each tree was identified to local name and, when possible, species. Within a 5×5 m subset of each plot, smaller plants including shrubs and vines were identified and the number of individual stems for each species counted (up to 20). Long-term feeding records of the Kasekela community were used to determine whether chimpanzees regularly consumed the fruits, leaves, or other parts of each plant species. A relational database (Microsoft Access) was used to calculate the mean basal area of chimpanzee food trees within each vegetation class in the range of each community.

For Mitumba and Kasekela, we estimated the community range for 2007 using the 99% minimum convex polygon (MCP) enclosing all observed locations with BIOTAS 1.01 (Ecological Software Solutions, LLC). Because the Kalande community is not habituated, information on ranging behavior was much more limited. We therefore used two estimates, the *minimal range* and the *likely range*. For the minimal range, we used 69 GPS locations where observers have seen Kalande chimpanzees (2002–2009), as well locations of three of the four intergroup encounters with the Kasekela chimpanzees recorded from 2000 on. To be conservative, we excluded one brief encounter with one lone female on 07 November 2003, as this encounter occurred further north than other encounters and may have involved an immigrating female outside of her normal range. Second, for the *likely range*, we included locations of chimpanzee nests found outside the park, adjacent to the Kalande range (2000–2006), and the locations of all 10 intergroup encounters between Kalande and Kasekela from 1998 on, including the 07 November 2003 encounter.

We then used ArcGIS 9.3 (ESRI) to join these ranges with a vegetation class layer to determine the total extent of each vegetation class for each community. The total basal area of chimpanzee food trees for each community was estimated as the sum of the extent of each vegetation class in each community times the mean basal area of chimpanzee food trees in that vegetation class in that community. The total number of stems of smaller food plants per community was likewise calculated.

Vegetation cover was derived using ERDAS, Inc software from a 4 m multispectral IKONOS satellite image acquired on July 30, 2000. The image was georeferenced using GPS ground control points and a digital elevation model. A 3×3 low-pass filter was applied and a Normalized Difference Vegetation Index (NDVI) was computed. Vegetation types were classified by identifying a range of NDVI values that represented a particular vegetation class using data collected on the ground in 2002 [45]. The accuracy of the vegetation map was estimated using 20×20 m vegetation plots, which revealed that 92% of evergreen forest/vine tangle plots and 70% of thicket woodland plots were correctly classified.

### Demographic Modeling

#### Critical prevalence

To determine the critical prevalence, we used a simple demographic calculation to show when, given fixed fertility and mortality rates, the prevalence of SIVcpz caused population decline. Let *f* be the fraction infected with SIVcpz. The baseline mortality rate is *μ* and SIVcpz positive chimpanzees have an increased mortality hazard given by the multiplier *ρ>1*. Annual fertility is *b* and the reduction in fertility from SIVcpz infection is given by *0<α≤1*. The annual rate of increase is the sum of average annual survivorship and annual fertility 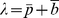 [e.g., 46]. Average survivorship and fertility are both mixtures for infected and uninfected rates with the mixing fraction given by *f*. An identity from survival analysis says that annual survival probability 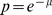. The annual rate of increase in our mixed population thus was:
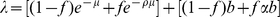
Setting *λ = 1*, we can solve for the prevalence, denoted f^*^, at which deterministic population stationarity is maintained. This value is:
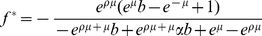
Whenever 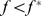, the average birth rate exceeds the death rate and the population increases (in the absence of stochasticity).

The average adult female mortality rate is approximately *μ* = 0.04. The interbirth interval for daughters is about 11 years and recruitment to age at first reproduction is approximately 50%. This makes “annual fertility” (which for this minimal model is a composite of fertility and recruitment) approximately *b* = 0.05.

#### Stochastic modeling

We employed a fully event-driven stochastic model of infection dynamics and demography. Using the direct method of Gillespie [47], we ran the event-driven model from the following master equations:
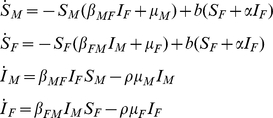
where 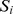 is the number of susceptible individuals of the *i*th sex (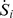 is its time derivative), 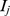 is the number of infected individuals of the *j*th sex (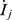 is its time derivative), 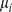 is the mortality rate of the *i*th sex, *b* is the birth rate (fertility times recruitment), 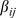 is the effective contact rate of transmission from sex *j* to sex *i*, 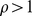 is the mortality multiplier (the degree to which SIVcpz infection increases annual mortality risk), and 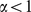 is the fertility multiplier (the degree to which SIVcpz infection decreases annual fertility). Migration does not appear in the master equations (though it does in the stochastic realizations) since the expected net migration is zero.

*Estimating the probability of SIVcpz transmission per coital act.* Preliminary evidence indicated that SIVcpz is transmitted primarily by sexual contact [2]. We estimated the effective contact rate [48], [49] for the epidemic model using the approach outlined by Morris [50]. Specifically, in a structured population, the effective contact rate for transmission from the *j*th class to the *i*th class in the model is
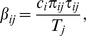
where 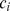 is the contact rate (i.e., number of copulations) of class *i*, 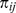 is the conditional probability that individuals of class *i* have contact with class *j* individuals (which for the case of a two-sex heterosexual model is 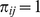 for all *i,j*), 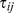 is the probability of transmission from *j* to *i* conditional on contact, and 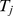 is the population size of class *j* individuals.

We used two approaches to estimate *τ*, the probability of transmission. First, given the possibility that SIVcpz transmission may occur at similar rates to HIV-1, we used the estimate of per-coital-act transmission probabilities for HIV-1 as determined by Gray and colleagues [23] in sero-discordant couples in Rakai, Uganda, using a generalized linear model with a complementary log-log-link. We used the unadjusted (overall) estimate of transmission, which is 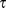 = 0.0011. Second, given the possibility that differences in mating patterns and physiology between humans and chimpanzees may result in different transmission rates, we estimated the probability of SIVcpz infection per coital act for Gombe chimpanzees. For a two-sex, completely heterosexual network, the basic reproduction number is the geometric mean of the off-diagonal elements of a 2×2 next generation matrix [48]. In particular,
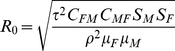
(1)where *τ* is the probability of transmission per coital act, *C_FM_* is the expected number of contacts of infectious males with susceptible females, *C_MF_* is the expected number of contacts of infectious females with susceptible males, *S_i_* is the expected number of susceptibles of the *i*th sex, *ρ* is the degree to which SIVcpz infection increases mortality, and *μ_i_* is the mortality rate of the *i*th sex. The epidemiological contact rate between individuals of sex *i* and sex *j* is given by Morris [50]:
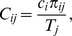
where *c_i_* is the expected number of matings for sex *i*, *π_ij_* is the conditional probability that, given an observed *i* mating, it will be with class *j* (obviously *π_ij_* = 1 for a purely heterosexual model like this), and *T_j_* is the population size of class *j*.

We solve equation 1 for *τ* yielding:
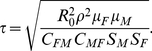
(2)We estimated these values using empirically determined SIVcpz prevalence, mating and mortality data from the habituated chimpanzees of the Kasekela community. We can estimate *R_0_* from the prevalence data using standard epidemiological theory [48], [49] as the inverse of the equilibrium fraction susceptible. The prevalence for the Kasekela community has remained remarkably stable for the years 2002 to 2009. We therefore used the median prevalence (10.8%) as an estimate of the current equilibrium fraction. This yielded *R_0_* = 1.21.

The number of susceptible individuals in the population was calculated based on the number of individuals of each sex that were observed copulating each calendar year. Individuals were considered susceptible if they were not infected at the start of the calendar year. All males that were 8 years old at the start of the calendar year, or who turned 8 during the calendar year, were included. At Gombe, males begin copulating with full intromission as early as four years old, and have been observed to ejaculate by age nine [51]. To be conservative, we included males starting at age eight, when testicular enlargement is usually first noticeable [51]. All females who had fully tumescent sexual swellings or who were recorded copulating during the calendar year were included.

We estimated *C_FM_* and *C_MF_* using behavioral data (2002–2007) to estimate copulation rates (copulation data from 2008–2009 are not yet available for analysis). Because we are interested in how frequently individuals copulate in years when they are sexually active, we included only years in which the individual was recorded mating at least once. We calculated each individual's annual copulation rate as the number of completed copulations recorded for that individual during his or her focal follows, divided by that individual's total observation time (in hours) as a focal subject for that year. We estimated the total number of copulations for each year by multiplying the hourly copulation rate by 12 hours of activity per day times 365.25 days per year (98.7% of all copulations recorded from 1976 through 2007 occurred between 6:00 am and 6:00 pm). We then took the mean of each individual's annual copulation rates to calculate the individual's overall copulation rate.

During the six-year study period, most males were sexually active for all six years (median = 6 years, range = 1 to 6 years). Males copulated a median 0.04/hr (range: 0.01–0.17/hr), or 209 times per year (range = 100–356/yr; n = 14 males). In contrast to males, female chimpanzees generally do not copulate when pregnant or lactating, and thus may not copulate at all for several years at a time, from the start of a pregnancy to the weaning of the infant. Thus, from 2002–2007, each female was sexually active during a median of one year (range = 1–5). Moreover, even in years in which females are sexually active, their copulation rates vary according to their ovarian cycle. Females generally copulate only during the 14 days of the 35-day cycle during which they have a fully tumescent sexual swelling [52]. Females may also copulate more frequently during conception cycles than nonconception cycles; Emery Thompson [52] found that parous females at two sites in Uganda copulated a median 0.40/hr in nonconceptive cycles (n = 18) and 0.85/hr in conceptive cycles. During focal follows in years in which they were sexually active, Gombe females copulated a median 0.41/hr (range: 0.083–1.0/hr; n = 21 females). This rate may be an overestimate, because females with full sexual swellings attract or are attracted to parties with many males, making it easier for observers to follow them. Assuming females copulated a this rate (0.41/hr) rate during the median 28.6% of days per year on which they had full swellings, females were estimated to have copulated a median 508 times per year.

The mortality multiplier, *ρ*, was previously estimated to be approximately 10 to 16 [2]. To err on the conservative side, we used 10 as our highest value. We also used *ρ* = 5, because this value lies towards the lower end of the 95% confidence interval for *ρ* [2]. Annual mortality was set at *μ_F_* = 0.04 for females and *μ_M_* = 0.05 for males, based on the mean of adult age-specific mortality for each sex [53]. Together, these values yielded *τ* = 0.0015 for *ρ* = 10, and *τ* = 0.00077 for *ρ* = 5.

*Intercommunity migration.* Both immigration and dispersal were incorporated into the stochastic model. A key question was whether migration could mitigate stochastic small-population effects of extinction, both of chimpanzee populations and of SIVcpz infections. It is expected that migration will rescue populations if there is more immigration than dispersal on average. However, in a closed collection of populations, the two will tend to balance. Consequently, we parameterized migration so that the expected net migration rate was zero. We modeled single populations with (i) emigration occurring at a rate proportional to the size of the female population and (ii) immigration occurring at fixed rates from outside the population.

Migration between communities is typically observed in adolescent females [51], although as noted in the Result section, older females also sometimes migrate. Including secondary transfers, females of known and estimated ages at Gombe had a mean age of migration of 15 years. Because our models are not age-structured, to parameterize migration, we followed a common practice for such models, by assuming that sojourn times are exponentially distributed with a rate parameter of the inverse of the mean time to event [54]. We thus parameterized the migration rate as 1/15 = 0.067. At Gombe, 30% of females who transferred when their infectious status was known were SIVcpz infected. SIVcpz prevalence rates at field sites in Cameroon have ranged from 5% to 35% [11], [55], suggesting that Gombe is towards the higher end of those prevalence rates. To model the range of likely conditions for chimpanzees, we thus ran both low (*p_F_* = 5%) and high (*p_F_* = 30%) probabilities that incoming females are infected with SIVcpz.

*Running simulations*. Simulations were constructed in Matlab using code modified from Keeling and Rohani [56]. We ran 10,000 replicate simulations, using the demographic parameters described above. Simulated populations were based on the observed number of sexually active individuals in Kasekela, which included all males age eight years and older, based on minimum age of producing ejaculate, and all females who were observed with fully tumescent sexual swellings and/or were observed copulating during that year. The number of initially infected males and females were taken as Poisson random numbers with rate parameters of λ = 1 and λ = 3, respectively. The initial number of susceptible individuals was then the difference between Poisson random numbers with rate parameters of λ_F_ = 18 and λ_M_ = 14 and the realized values of initially infected females and males.

We ran simulations under 12 sets of different starting conditions, in which we varied the following parameters: migration (with or without); *τ*, the probability of transmission per coital act (high, *τ* = 0.0015, medium, *τ* = 0.0011, and low, *τ* = 0.0008, with the high and low values based on SIVcpz data and the medium value based on HIV-1 data), *ρ* (high, *ρ* = 10, or low, *ρ* = 5), and when migration was included, with probabilities that immigrating females would be infected of 5% and 30%. In the estimates derived from SIVcpz data, the value of *τ* varies directly with the value for *ρ*. In contrast, the estimate derived from HIV-1 data is independently derived, so we ran simulations with both high and low values of *ρ* with that estimate of *τ*.

### Accession Numbers

Newly derived SIVcpz sequences have been deposited in GenBank under accession numbers GU992204 and GU992205.

## Supporting Information

Figure S1 Prospective epidemiological study of SIVcpz infection in three Gombe communities. SIVcpz infection rates were determined for Kalande (KL), Kasekela (KK), and Mitumba (MT) communities in 6-month intervals from January 2002 to December 2009. Presence (“1”) or absence (“0”) of SIVcpz infection is indicated, as determined by fecal (and/or urine for Kasekela and Mitumba) testing. Each chimpanzee is listed in its community of residence at the start of each sampling period. Previously published data are shown in green (light green fields indicate actual data; dark green fields indicate inferred data as reported [2]), while new data are shown in yellow (light yellow fields indicate actual data; dark yellow fields indicate inferred data; see Methods). For chimpanzees who died or disappeared, the Date Of Death (DOD) or Date Last Seen (DLS) is listed. Kalande chimpanzees, who are presumed to be dead, are also listed (see Tables S3 and S4 for more information). The total number of individuals tested and found to be SIVcpz infected is shown beneath each community. A previously fecal antibody positive infant (Ch-103), who became antibody negative after weaning (and is thus free of SIVcpz infection), is denoted by an asterisk.
(0.21 MB PDF)

Table S1 Non-invasive testing of Kalande chimpanzees for SIVcpz infection.
(0.10 MB PDF)

Table S2 Non-invasive testing of Mitumba and Kasekela chimpanzees for SIVcpz infection.
(0.07 MB PDF)

Table S3 Kalande demographics.
(0.07 MB PDF)

Table S4 Evidence for existence, departure, and causes of death for Kalande chimpanzees.
(0.05 MB PDF)

Table S5 Genetic evidence for dyadic relationships.
(0.11 MB PDF)

Table S6 Genetic evidence for paternal relationships.
(0.06 MB PDF)

## References

[1] Silvestri G (2008). Immunity in natural SIV infections.. J Intern Med.

[2] Keele BF, Jones JH, Terio KA, Estes JD, Rudicell RS (2009). Increased mortality and AIDS-like immunopathology in wild chimpanzees infected with SIVcpz.. Nature.

[3] Walsh PD, Abernethy KA, Bermejo M, Beyers R, De Wachter P (2003). Catastrophic ape decline in western equatorial Africa.. Nature.

[4] Campbell G, Kuehl H, N'Goran Kouamé P, Boesch C (2008). Alarming decline of West African chimpanzees in Côte d'Ivoire.. Curr Biol.

[5] Köndgen S, Kühl H, N'Goran PK, Walsh PD, Schenk SS (2008). Pandemic human viruses cause decline of endangered great apes.. Curr Biol.

[6] Hahn BH, Shaw GM, De Cock KM, Sharp PM (2000). AIDS as a zoonosis: scientific and public health implications.. Science.

[7] Aghokeng AF, Ayouba A, Mpoudi Ngole E, Loul S, Liegeois F (2010). Extensive survey on the prevalence and genetic diversity of SIVs in primate bushmeat provides insights into risks for potential new cross-species transmissions.. Infect Genet Evol.

[8] Phillips-Conroy JE, Jolly CJ, Petros B, Allan JS, Desrosiers RC (1994). Sexual transmission of SIVagm in wild grivet monkeys.. J Med Primatol.

[9] Santiago ML, Range F, Keele BF, Li YY, Bailes E (2005). Simian immunodeficiency virus infection in free-ranging sooty Mangabeys (Cercocebus atys atys) from the Tai Forest, Cote d'Ivoire: Implications for the origin of epidemic human immunodeficiency virus type 2.. J Virol.

[10] Santiago ML, Lukasik M, Kamenya S, Li Y, Bibollet-Ruche F (2003). Foci of endemic simian immunodeficiency virus infection in wild-living eastern chimpanzees (Pan troglodytes schweinfurthii).. J Virol.

[11] Keele BF, Van Heuverswyn F, Li Y, Bailes E, Takehisa J (2006). Chimpanzee reservoirs of pandemic and nonpandemic HIV-1.. Science.

[12] Li Y, Ndjango J-B, Learn GH, Robertson J, Takehisa J (2010). Molecular epidemiology of simian immunodeficiency virus in eastern chimpanzees and gorillas..

[13] Worobey M, Santiago ML, Keele BF, Ndjango JBN, Joy JB (2004). Origin of AIDS - Contaminated polio vaccine theory refuted.. Nature.

[14] Sharp PM, Shaw GM, Hahn BH (2005). Simian immunodeficiency virus infection of chimpanzees.. J Virol.

[15] Pusey AE, Pintea LP, Wilson ML, Kamenya S, Goodall J (2007). The contribution of long-term research at Gombe National Park to chimpanzee conservation.. Conserv Biol.

[16] Greengrass E (2000). The sudden decline of a community of chimpanzees at Gombe National Park.. Pan Africa News.

[17] Goodall J (1986). The Chimpanzees of Gombe: Patterns of Behavior.

[18] Pusey AE, Wilson ML, Collins DA (2008). Human impacts, disease risk, and population dynamics in the chimpanzees of Gombe National Park, Tanzania.. Am J Primatol.

[19] Santiago ML, Rodenburg CM, Kamenya S, Bibollet-Ruche F, Gao F (2002). SIVcpz in wild chimpanzees.. Science.

[20] Pusey AE, Hamburg DA, McCown ER (1979). Intercommunity transfer of chimpanzees in Gombe National Park. The Great Apes.

[21] Husch B, Beers TW, Kershaw JA (2003). Forest Mensuration.

[22] Lacy RC (1993). Vortex - a computer-simulation model for population viability analysis.. Wildl Res.

[23] Gray RH, Wawer MJ, Brookmeyer R, Sewankambo NK, Serwadda D (2001). Probability of HIV-1 transmission per coital act in monogamous, heterosexual, HIV-1-discordant couples in Rakai, Uganda.. Lancet.

[24] Wrangham RW, Wilson ML, Muller MN (2006). Comparative rates of violence in chimpanzees and humans.. Primates.

[25] Mitani JC, Watts DP, Muller MN (2002). Recent developments in the study of wild chimpanzee behavior.. Evol Anthropol.

[26] Nishida T, Kawanaka K (1985). Within-group cannibalism by adult male chimpanzees.. Primates.

[27] Wilson ML, Wrangham RW (2003). Intergroup relations in chimpanzees.. Annu Rev Anthropol.

[28] Nishida T, Hiraiwa-Hasegawa M, Hasegawa T, Takahata Y (1985). Group extinction and female transfer in wild chimpanzees in the Mahale National Park, Tanzania.. Z Tierpsychol.

[29] van der Paal L, Shafer LA, Todd J, Mayanja BN, Whitworth JAG (2007). HIV-1 disease progression and mortality before the introduction of highly active antiretroviral therapy in rural Uganda.. AIDS.

[30] Hladik F, McElrath MJ (2008). Setting the stage: host invasion by HIV.. Nat Rev.

[31] Morin PA, Chambers KE, Boesch C, Vigilant L (2001). Quantitative polymerase chain reaction analysis of DNA from noninvasive samples for accurate microsatellite genotyping of wild chimpanzees (*Pan troglodytes verus*).. Mol Ecol.

[32] Constable JL, Ashley MV, Goodall J, Pusey AE (2001). Noninvasive paternity assignment in Gombe chimpanzees.. Mol Ecol.

[33] Wroblewski EE, Murray CM, Keele BF, Schumacher-Stankey J, Hahn BH (2009). Male dominance rank and reproductive success in chimpanzees, *Pan troglodytes schweinfurthii*.. Anim Behav.

[34] Marshall TC, Slate J, Kruuk LEB, Pemberton JM (1998). Statistical confidence for likelihood-based paternity inference in natural populations.. Mol Ecol.

[35] Goodnight KF, Queller DC (1999). Computer software for performing likelihood tests of pedigree relationship using genetic markers.. Mol Ecol.

[36] Queller DC, Goodnight KF (1989). Estimating relatedness using genetic-markers.. Evolution.

[37] Larkin MA, Blackshields G, Brown NP, Chenna R, McGettigan PA (2007). Clustal W and clustal X version 2.0.. Bioinformatics.

[38] Ronquist F, Huelsenbeck JP (2003). MrBayes 3:Bayesian phylogenetic inference under mixed models.. Bioinformatics.

[39] Plumptre AJ, Reynolds V (1996). Censusing chimpanzees in the Budongo forest, Uganda.. Int J Primatol.

[40] Thomas L, Buckland ST, Rexstad EA, Laake JL, Strindberg S (2010). Distance software: design and analysis of distance sampling surveys for estimating population size.. J Appl Ecol.

[41] Plumptre AJ, Reynolds V (1997). Nesting behavior of chimpanzees: Implications for censuses.. Intern J Primatol.

[42] Plumptre AJ (2000). Monitoring mammal populations with line transect techniques in African forests.. J Appl Ecol.

[43] Wilson ML, Wallauer W, Pusey AE (2004). New cases of intergroup violence among chimpanzees in Gombe National Park, Tanzania.. Int J Primatol.

[44] Murray CM, Eberly LE, Pusey AE (2006). Foraging strategies as a function of season and rank among wild female chimpanzees (*Pan troglodytes*).. Behav Ecol.

[45] Pintea L (2007). Applying satellite imagery and GIS for chimpanzee habitat change detection and conservation [PhD Thesis].

[46] Schaffer WM (1974). Optimal reproductive effort in fluctuating environments.. Am Nat.

[47] Gillespie DT (1977). Exact stochastic simulation of coupled chemical reactions.. J Phys Chem.

[48] Diekmann O, Heesterbeek H (2000). Mathematical epidemiology of infectious diseases: Model building, analysis and interpretation.

[49] Anderson RM, May RM (1991). Infectious Diseases of Humans: Dynamics and Control.

[50] Morris M (1991). A log-linear modeling framework for selective mixing.. Math Biosci.

[51] Pusey AE (1990). Behavioural changes at adolescence in chimpanzees.. Behaviour.

[52] Emery Thompson M (2005). Reproductive endocrinology of wild chimpanzees (Pan troglodytes schweinfurthii): methodological considerations and the role of hormones in sex and conception.. Am J Primatol.

[53] Hill K, Boesch C, Goodall J, Pusey AE, Williams JM, Wrangham RW (2001). Mortality rates among wild chimpanzees.. J Hum Evol.

[54] Caswell H (2001). Matrix Population Models: Construction, Analysis and Interpretation.

[55] Van Heuverswyn F, Li Y, Bailes E, Neel C, Lafay B (2007). Genetic diversity and phylogeographic clustering of SIVcpzPtt in wild chimpanzees in Cameroon.. Virology.

[56] Keeling MJ, Rohani P (2007). Modeling Infectious Diseases in Humans and Animals.

