# Supplementary material for: Impact of Simian Immunodeficiency Virus Infection on Chimpanzee Population Dynamics
Source: PLoS Pathog. 2010 Sep 23;6(9):e1001116. doi: 10.1371/journal.ppat.1001116 (PMC2944804; doi:10.1371/journal.ppat.1001116)
Supplement: Figure S1 — Prospective epidemiological study of SIVcpz infection in three Gombe communities. SIVcpz infection rates were determined for Kalande (KL), Kasekela (KK), and Mitumba (MT) communities in 6-month intervals from January 2002 to December 2009. Presence (“1”) or absence (“0”) of SIVcpz infection is indicated, as determined by fecal (and/or urine for Kasekela and Mitumba) testing. Each chimpanzee is listed in its community of residence at the start of each sampling period. Previously published data are shown in green (light green fields indicate actual data; dark green fields indicate inferred data as reported [2]), while new data are shown in yellow (light yellow fields indicate actual data; dark yellow fields indicate inferred data; see Methods). For chimpanzees who died or disappeared, the Date Of Death (DOD) or Date Last Seen (DLS) is listed. Kalande chimpanzees, who are presumed to be dead, are also listed (see Tables S3 and S4 for more information). The total number of individuals tested and found to be SIVcpz infected is shown beneath each community. A previously fecal antibody positive infant (Ch-103), who became antibody negative after weaning (and is thus free of SIVcpz infection), is denoted by an asterisk. (0.21 MB PDF) [file ppat.1001116.s001.pdf]

Community	Ape ID	Sex	2002 (Jan-Jun)	2002 (Jul-Dec)	2003 (Jan-Jun)	2003 (Jul-Dec)	2004 (Jan-Jun)	2004 (Jul-Dec)	2005 (Jan-Jun)	2005 (Jul-Dec)	2006 (Jan-Jun)	2006 (Jul-Dec)	2007 (Jan-Jun)	2007 (Jul-Dec)	2008 (Jan-Jun)	2008 (Jul-Dec)	2009 (Jan-Jun)	2009 (Jul-Dec)	Death/Emigration
Kalande	Ch-064	F	1	1	1	1	1	1	1	1	1	1	1	1	1	1	1	1	
	Ch-070	F	1																Presumed dead
	Ch-071	F	1	1	1	1	1	1	1										Emigrated to KK on 15-Feb-05
	Ch-081	M	0	0															Presumed dead
	Ch-082	F		1															Presumed dead
	Ch-083	F	0																Presumed dead
	Ch-084	F			0														Presumed dead
	Ch-085	M	0	0															Presumed dead
	Ch-086	F	1	1	1	1	1	1	1	1	1	1	1	1	1	1	1	1	
	Ch-087	M	0																DOD 13-Aug-02
	Ch-088	F					0	0	0	0	0	0	0	0	0	0	0	0	
	Ch-089	F		0						1	1	1	1	1					
	Ch-091	F					1												Presumed dead
	Ch-092	F			0														Presumed dead
	Ch-093	F			0	0	0	0	0	0	0	0	0	0	0	0	0	0	
	Ch-095	F						0											Presumed dead
	Ch-098	F	0	0	0	0	0												Emigrated to MT on 08-Jun-04
	Ch-099	F				1													Emigrated to KK on 15-Sep-03
	Ch-100	M						0	0	0	0	0	0	1	1	1	1	1	
	Ch-106	F										1	1	1	1	1	1		
	Ch-107	F								1									Presumed dead
	Ch-108	F							0	0	0	0	0	0	0	0	0	0	
	Ch-109	F								0	0	0	0	0	0	0	0	0	
	Ch-110	M									0	0	0	0	0	0	0	0	
	Ch-118	M														1			
	Ch-121	F																1	
Number of individuals sampled			9	8	7	6	7	7	7	9	9	10	10	10	9	10	9	9	
Number of SIVcpz infected individuals			4	4	3	4	4	3	3	4	3	4	4	5	4	5	4	4	
Kasekela	Ch-001	F	0	0	0	0	0	0	0	0	0	0	0	0	0	0	0	0	
	Ch-002	F	0	0	0	0	0	0	0	0	0	0	0	0	0	0	0	0	
	Ch-003	M	0	0	0	0	0	0	0	0	0	0	0	0	0	0	0	0	
	Ch-004	M	0	0	0	0	0	0	0	0		1	1	1	1	1	1	1	
	Ch-005	M	0	0	0	0	0	0	0	0	0	0	0	0	0	0	0	0	
	Ch-006	M	1	1	1	1	1	1	1	1	1	1	1						DLS 23-Jan-07
	Ch-007	M	0	0	0	0	0	0	0	0	0	0	0	0	0	0	0	0	
	Ch-008	M	0																DLS 06-Nov-02
	Ch-009	F	0	0	0	0	0	0	0	0	0	0	0	0	0	0	0	0	
	Ch-010	M	0	0	0	0	0	0	0	0	0	0	0	0	0	0	0	0	
	Ch-011	F	0	0	0	0	0	0											DLS 01-Sep-04
	Ch-012	F	0	0	0	0	0	0	0	0	0	0	0	0	0	0	0	0	
	Ch-013	M	0	0	0	0	0	0	0	0	0	0	0	0	0	0	0	0	
	Ch-014	M		0	0	0	0	0	0	0	0	0	0	0	0	0	0	0	
	Ch-015	F	0	0	0	0	0	0	0	0	0	0	0	0	0	0	0	0	
	Ch-016	M	0	0	0	0	0	0											DOD 24-Aug-04
	Ch-017	F	0	0	0	0	0	0	0	0	0	0	0	0	0	0	0	0	
	Ch-018	F	0	0	0	0	0	0	0	0	0	0	0	0	0	0	0	0	
	Ch-019	F	0	0	0	0	0	0	0	0	0	0	0	0	0	0	0	0	
	Ch-020	M	0	0	0	0	0	0	0	0	0	0	0	0	0	0	0	0	
	Ch-021	F		0	0	0	0	0	1	1	1	1	1	1	1	1	1	1	DOD 04-Jan-10
	Ch-022	F	1	1	1	1	1	1	1	1	1	1	1	1	1	1	1	1	
	Ch-023	M	0	0	0	0	0	0	0	0	0	0	0	0	0	0	0	0	
	Ch-024	F	0	0	0	0	0	0											DOD 03-Oct-05
	Ch-025	F	0	0	0	0	0	0	0	0	0	0	0	0	0	0	0	0	
	Ch-026	F	0	0	0	0	0	0	0	0	0	0	0	0	0	0	0	0	
	Ch-027	M	0	0	0	0	0	0	0	0	0	0	0	0	0	0	0	0	
	Ch-028	F	0	0	0	0	0	0	0	0									DOD 5-Nov-06
	Ch-029	F	0	0	0	0	0	0	0	0	0	0	0	0	0	0	0	0	
	Ch-030	F	1	1	1	1													DLS 08-Dec-03
	Ch-031	F	0	0	0	0	0	0	0	0	0	0	0	0	0	0	0	0	
	Ch-032	F	0	0	0	0	0	0	0	0	0	0	0	0	0	0	0	0	
	Ch-033	F	1	1	1	1	1	1	1	1	1	1	1	1	1				DLS 02-Mar-08
	Ch-034	M	0	0	0	0	0	0	0	0	0	0	0	0	0	0	0	0	
	Ch-035	M	0	0	0	0	0	0	0	0	0	0	0	0	0	0	0	0	
	Ch-036	F	0	0	0	0	0	0			1	1	1	1					DOD 07-Nov-07
	Ch-041	F	0	0	0	0	0	0	0	0	0	0	0	0					DLS 02-Oct-07
	Ch-050	F	0	0	0	0	0	0	0	0	0	0	0	0	0	0	0	0	
	Ch-051	M	0	0	0	0	0	0	0	0	0	0	0	0	0	0	0	0	
	Ch-052	M	0	0	0	0	0	0		1	1	1	1	1	1	1	1	1	
	Ch-053	F	0	0	0	0	0	0	0	0	0	0	0	0	0	0	0	0	
	Ch-054	M	0	0	0	0	0	0	0	0	0	0	0	0	0	0	0	0	
	Ch-055	F	0	0	0	0	0	0	0	0	0	0	0	0	0	0	0	0	
	Ch-056	M	0	0	0	0	0	0	0	0	0	0	0	0	0	0	0	0	
	Ch-057	F	0	0	0	0	0	0	0	0	0	0	0	0	0	0	0	0	
	Ch-058	M	0	0	0	0	0	0	0	0	0	0	0	0	0	0	0	0	
	Ch-060	M		0	0	0	0	0	0	0	0	0	0	0	0	0	0	0	
	Ch-061	F		0	0	0	0	0	0	0	0	0	0	0	0	0	0	0	
	Ch-062	M		1	1	1	1												DOD 13-Jun-04
	Ch-071	F								1	1	1	1	1	1	1	1	1	
	Ch-072	M									0								DOD 18-Nov-06
	Ch-073	F						0	0	0	0	0	0	0	0	0	0	0	
	Ch-077	M							0	0	0	0	0	0	0	0	0	0	
	Ch-078	F	0	0	0	0	0	0	0	0	0	0	0	0	0	0	0	0	
	Ch-079	F		0	0	0	0	0	0	0	0	0	0	0	0	0	0	0	
	Ch-080	F								1	1	1	1	1	1	1	1	1	
	Ch-090	M											0	0	0	0	0	0	
	Ch-096	F																0	
	Ch-097	F											0	0	0	0			
	Ch-099	F					1	1	1	1	1	1							DOD 13-Nov-06
	Ch-101	F					0	0	0	0	0	0	0	0	0	0	0	0	
	Ch-102	F						0	0	0	0	0	0	0	0	0	0	0	
	Ch-103*	F										(1*)	0	0	0	0	0	0	
	Ch-104	F										0	0	0					DLS 02-Oct-07
	Ch-105	F						0	0	0	0	0	0	0	0	0	0	0	
	Ch-114	F											0	0	0	0	0	0	
	Ch-115	M											0	0	0	0	0	0	
	Ch-116	M														0	0	0	
	Ch-117	F												0	0	0	0	0	
	Ch-119	M															0		
	Ch-120	F															0	0	
	Ch-123	F																0	
Number of individuals sampled			45	50	50	50	51	53	49	52	52	54	57	57	54	54	55	56	
Number of SIVcpz infected individuals			4	5	5	5	5	4	5	8	9	10	9	8	7	6	6	6	
Mitumba	Ch-038	F	0	0															DLS 25-Jun-03
	Ch-039	F	0	0			1	1	1	1	1	1	1	1	1	1	1	1	
	Ch-042	F	0	0	0	0	0	0	0	0	0	0	0	0	0	0	0	0	
	Ch-044	F	0	0	0	0	0	0	0	0	0	0	0	0	0	0	0	0	
	Ch-045	M	1	1	1	1	1	1											DOD 22-Dec-04
	Ch-046	F	0	0	0	0	0	0	0	0	0	0	0	0	0	0	0	0	
	Ch-047	F	0	0	0	0	0	0	0	0	0	0	0	0	0	0	0	0	
	Ch-048	M	0			1	1	1	1	1	1	1	1	1	1	1	1	1	
	Ch-049	F		0	0	0	0	0	0	0	0	0	0	0	0	0	0	0	
	Ch-059	M				0	0	0	0	0	0	0	0	0	0	0	0	0	
	Ch-063	M						0	0	0	0	0	0	0	0	0	0	0	
	Ch-065	M	0	0	0	0	0	0	0	0	0	0	0	0	0	0	0	0	
	Ch-066	F	0	0	0	0	0	0	0	0	0	0	0	0	0	0	0	0	
	Ch-067	M		0	0	0	0	0	0	0	0	0	0	0	0	0	0	0	
	Ch-068	M		0	0	0	0	0	0	0	0	0	0	0	0	0	0	0	
	Ch-069	M		0	0	0	0	0											DOD 17-Jan-05
	Ch-075	M							0	0	0	0	0	0	0	0	0	0	
	Ch-076	F									0	0	0	0	0	0	0	0	
	Ch-080	F					1	1	1										Emigrated to KK on 15-Feb-05
	Ch-094	F													0	0	0	0	
	Ch-096	F				0	0	0	0	0	0	0	0	0	0	0	0		Emigrated to KK on 16-Jun-09
	Ch-098	F						0	0	0	0	0	0	0	0	0	0	0	
	Ch-111	F												0	0	0	0	0	
	Ch-112	F														0	0	0	
	Ch-113	M													0	0	0	0	
	Ch-122	F																0	
Number of individuals sampled			10	13	11	14	16	18	17	16	17	17	17	18	20	21	21	21	
Number of SIVcpz infected individuals			1	1	1	2	4	4	3	2	2	2	2	2	2	2	2	2	

Figure S1. Prospective epidemiological study of SIVcpz infection in three Gombe communities. SIVcpz infection rates were determined for Kalande (KL), Kasekela (KK), and Mitumba (MT) communities in 6-month intervals from January 2002 to December 2009. Presence ("1") or absence ("0") of SIVcpz infection is indicated, as determined by fecal (and/or urine for Kasekela and Mitumba) testing. Each chimpanzee is listed in its community of residence at the start of each sampling period. Previously published data are shown in green (light green fields indicate actual data; dark green fields indicate inferred data as reported [2]), while new data are shown in yellow (light yellow fields indicate actual data; dark yellow fields indicate inferred data; see Methods). For chimpanzees who died or disappeared, the Date Of Death (DOD) or Date Last Seen (DLS) is listed. Kalande chimpanzees, who are presumed to be dead, are also listed (see Tables S3 and S4 for more information). The total number of individuals tested and found to be SIVcpz infected is shown beneath each community. A previously fecal antibody positive infant (Ch-103), who became antibody negative after weaning (and is thus free of SIVcpz infection), is denoted by an asterisk.
