# Supplementary material for: Impact of Simian Immunodeficiency Virus Infection on Chimpanzee Population Dynamics
Source: PLoS Pathog. 2010 Sep 23;6(9):e1001116. doi: 10.1371/journal.ppat.1001116 (PMC2944804; doi:10.1371/journal.ppat.1001116)
Supplement: Table S1 — Non-invasive testing of Kalande chimpanzees for SIVcpz infection. (0.10 MB PDF) [file ppat.1001116.s002.pdf]

Table S1. Non-invasive testing of Kalande chimpanzees for SIVcpz infection.

Individual[1]	Sex[2]	Comm-unity[3]	Sample Code	Collection Date[4]	SIVcpz fecal WB[5]	SIVcpz fecal vRNA	Genbank Accession numbers[6]			SIVcpz strain	mtDNA haplotype[7]	Microsatellite Loci							
							Full Length	pol	env			D18s536	D4s243	D10s676	D9s922	D2s1326	D2s1333	D4s1627	D9s905
Ch-064	F	KL	39	11-Nov-01	pos	pos	*DQ374657*			TAN2	13	141/161	200/204	182/190	298/302				
		KL	229	17-Mar-02	pos	neg					13	141/161	200/204	182/190	298/302				
		KL	281	24-Dec-02	pos	neg					13	141/161	200/204	182/190	298/302				
		KL	274	20-Jan-03	pos	neg					13	141/161	200/204	182/190	298/302				
		KL	257	24-Jan-03	pos	neg					13	141/161	200/204	182/190	298/302				
		KL	273	24-Jan-03	pos	neg					13	141/161	200/204	182/190	298/302				
		KL	297	29-Jul-03	pos	neg					13	141/161	200/204	182/190	298/302				
		KL	228	28-Aug-03	pos	neg					13	141/161	200/204	182/190	298/302				
		KL	308	26-Sep-03	pos	neg					13	141/161	200/204	182/190	298/302				
		KL	301	9-Oct-03	pos	pos					13	141/161	200/204	182/190	298/302				
		KL	365	30-Jan-04	pos	neg					13	141/161	200/204	182/190	298/302				
		KL	368	10-Feb-04	pos	neg					13	141/161	200/204	182/190	298/302				
		KL	529	28-Jul-04	pos	pos					13	141/161	200/204	182/190	298/302				
		KL	536	4-Aug-04	pos	neg					13	141/161	200/204	182/190	298/302				
		KL	546	13-Aug-04	pos	pos					13	141/161	200/204	182/190	298/302				
		KL	547b	13-Aug-04	pos	pos					13	141/161	200/204	182/190	298/302				
		KL	568	14-Sep-04	pos	pos					13	141/161	200/204	182/190	298/302	**256/260**	**322/326**	**229/237**	**287/295**
		KL	569	14-Sep-04	pos	pos					13	141/161	200/204	182/190	298/302				
		KL	570	14-Sep-04	pos	pos					13	141/161	200/204	182/190	298/302				
		KL	579	4-Oct-04	pos	neg					13	141/161	200/204	182/190	298/302	**256/260**	**322/326**	**229/237**	**287/295**
		KL	580	4-Oct-04	pos	pos					13	141/161	200/204	182/190	298/302				
		KL	601	8-Dec-04	pos	neg					13	141/161	200/204	182/190	298/302	**256/260**	**322/326**	**229/237**	**287/295**
		KL	918	20-Dec-04	pos						13	141/161	200/204	182/190	298/302				
		KL	769	12-Mar-05	pos						13	141/161	200/204	182/190	298/302				
		KL	770	12-Mar-05	pos						13	141/161	200/204	182/190	298/302				
		KL	771	12-Mar-05	pos						13	141/161	200/204	182/190	298/302				
		KL	773	21-Mar-05	pos						13	141/161	200/204	182/190	298/302				
		KL	774	23-Mar-05	pos						13	141/161	200/204	182/190	298/302				
		KL	775	23-Mar-05	pos						13	141/161	200/204	182/190	298/302				
		KL	777	29-Mar-05	pos						13	141/161	200/204	182/190	298/302				
		KL	778	29-Mar-05	pos						13	141/161	200/204	182/190	298/302				
		KL	870	17-Jun-05	pos						13	141/161	200/204	182/190	298/302				
		KL	874	17-Jun-05	pos						13	141/161	200/204	182/190	298/302				
		KL	880	24-Jul-05	pos						13	141/161	200/204	182/190	298/302				
		KL	888	13-Aug-05	pos						13	141/161	200/204	182/190	298/302				
		KL	890	13-Aug-05	pos						13	141/161	200/204	182/190	298/302				
		KL	914	3-Nov-05	pos						13	141/161	200/204	182/190	298/302				
		KL	935	5-Feb-06	pos						13	141/161	200/204	182/190	298/302				
		KL	947	3-Apr-06	pos						13	141/161	200/204	182/190	298/302				
		KL	945	13-May-06	pos						13	141/161	200/204	182/190	298/302				
		KL	1129	6-Jun-06	pos						13	141/161	200/204	182/190	298/302				
		KL	1130	6-Jun-06	pos						13	141/161	200/204	182/190	298/302				
		KL	1134	16-Jul-06	pos						13	141/161	200/204	182/190	298/302				
		KL	1135	22-Jul-06	pos						13	141/161	200/204	182/190	298/302				
		KL	1136	22-Jul-06	pos						13	141/161	200/204	182/190	298/302				
		KL	1148	1-Aug-06	pos						13	141/161	200/204	182/190	298/302				
		KL	1149	1-Aug-06	pos						13	141/161	200/204	182/190	298/302				
		KL	1143	13-Aug-06	pos						13	141/161	200/204	182/190	298/302				
		KL	1146	29-Aug-06	pos						13	141/161	200/204	182/190	298/302				
		KL	1288	9-Jul-07	pos							141/161	200/204	182/190	298/302				
		KL	1405	9-Jul-07	pos						13	141/161	200/204	182/190	298/302				
		KL	1287	8-Aug-07	pos							141/161	200/204	182/190	298/302				
		KL	1298	18-Aug-07	pos							141/161	200/204	182/190	298/302				
		KL	1289	9-Sep-07	pos							141/161	200/204	182/190	298/302				
		KL	1291	9-Sep-07	pos							141/161	200/204	182/190	298/302				
		KL	1292	9-Sep-07	pos							141/161	200/204	182/190	298/302				
		KL	1301	23-Sep-07	pos							141/161	200/204	182/190	298/302				
		KL	1302	23-Sep-07	pos							141/161	200/204	182/190	298/302				
		KL	1280	6-Dec-07	pos							141/161	200/204	182/190	298/302				
		KL	1281	6-Dec-07	pos							141/161	200/204	182/190	298/302				
		KL	1376	9-Jun-08	pos							141/161	200/204	182/190	298/302				
		KL	1481	29-Jul-08	pos							141/161	200/204	182/190	298/302				
		KL	1523	29-Jul-08	pos							141/161	200/204	182/190	298/302				
		KL	1526	9-Aug-08	pos							141/161	200/204	182/190	298/302				
		KL	1433	11-Aug-08	pos							141/161	200/204	182/190	298/302				
		KL	1530	11-Aug-08	pos							141/161	200/204	182/190	298/302				
		KL	1441	1-Sep-08	pos							141/161	200/204	182/190	298/302				
		KL	1469	17-Sep-08	pos							141/161	200/204	182/190	298/302				
		KL	1532	17-Sep-08	pos							141/161	200/204	182/190	298/302				
		**KL**	**1585**	**16-Jun-09**	**pos**						**13**	**141/161**	**200/204**	**182/190**	**298/302**				
		**KL**	**1586**	**25-Jun-09**	**pos**						**13**	**141/161**	**200/204**	**182/190**	**298/302**	**256/260**	**322/326**	**229/237**	**287/295**
		**KL**	**1728**	**28-Jul-09**	**pos**						**13**	**141/161**	**200/204**	**182/190**	**298/302**				
		**KL**	**1592**	**11-Aug-09**	**pos**						**13**	**141/161**	**200/204**	**182/190**	**298/302**				
		**KL**	**1618**	**24-Aug-09**	**pos**						**13**	**141/161**	**200/204**	**182/190**	**298/302**				
		KL	283	na	pos	neg					13	141/161	200/204	182/190	298/302				
Ch-070	F	KL	239	13-Mar-02	pos	pos		*AY1819891*		TAN4	2	141/141	200/204	182/190	268/302	**256/260**	**302/302**	**233/237**	**295/295**
Ch-071	F	KL	242	22-Jun-02	pos	pos			*AY181993*	TAN5	10	141/173	196/196	190/190	290/302	**244/248**	**318/322**	**225/229**	**279/287**
		KL	298	17-Aug-03	pos						10	141/173	196/196	190/190	290/302	**244/248**	**318/322**	**225/229**	**279/287**
		KL	367	1-Jun-04	pos	neg					10	141/173	196/196	190/190	290/302	**244/248**	**318/322**	**225/229**	**279/287**
		KL	597	28-Nov-04	pos	neg					10	141/173	196/196	190/190	290/302	**244/248**	**318/322**	**225/229**	**279/287**
		KK	734	1-Mar-05	pos	pos		*FJ895394*		TAN5	10	141/173	196/196	190/190	290/302	**244/248**	**318/322**	**225/229**	**279/287**
		KK	989	22-Jan-06	pos	pos			*FJ895382*	TAN5	10	141/173	196/196	190/190	290/302				
		KK	1167	12-Feb-07	pos							141/173	196/196	190/190	290/302				
		KK	1323	7-Apr-07	pos							141/173	196/196	190/190	290/302				
		KK	1399	9-May-08	pos							141/173	196/196	190/190	290/302				
		KK	1508	15-Oct-08	pos							141/173	196/196	190/190	290/302				
		**KK**	**1597**	**10-Aug-09**	**pos**						**10**	**141/173**	**196/196**	**190/190**	**290/302**				**279/287**
		**KK**	**1712**	**6-Oct-09**	**pos**						**10**	**141/173**	**196/196**	**190/190**	**290/302**				
**Ch-081**	**M**	**KL**	**251**	**14-Apr-02**	**neg**						**1**	**141/161**	**196/204**	**158/182**	**290/294**	**244/260**	**302/318**	**233/237**	**279/295**
		**KL**	**233**	**24-Aug-02**	**neg**						**1**	**141/161**	**196/204**	**158/182**	**290/294**	**244/260**	**302/318**	**233/237**	**279/295**
Ch-082	F	KL	244	7-Aug-02	pos	neg					1	141/141	204/204	186/190	302/306		**310/322**	**237/249**	**271/295**
		KL	234	22-Aug-02	pos	pos		*FJ895402*		TAN19	1	141/141	204/204	186/190	302/306	**248/260**	**310/322**	**237/249**	**271/295**
**Ch-083**	**F**	**KL**	**241**	**15-Mar-02**	**neg**						**1**	**141/141**	**231/231**	**186/186**	**294/302**	**236/248**	**310/322**	**237/249**	**271/287**
**Ch-084**	**F**	**KL**	**302**	**18-Apr-03**	**neg**						**13**	**141/161**	**200/235**	**182/190**	**298/302**	**244/256**	**302/318**	**237/241**	**279/295**
**Ch-085**	**M**	**KL**	**231**	**15-Mar-02**	**neg**						**2**	**141/161**	**196/196**	**186/190**	**294/302**	**244/256**	**318/322**	**225/241**	**287/287**
		**KL**	**236**	**14-Apr-02**	**neg**						**2**	**141/161**	**196/196**	**186/190**	**294/302**	**244/256**	**318/322**	**225/241**	**287/287**
		**KL**	**247**	**28-May-02**	**neg**						**2**	**141/161**	**196/196**	**186/190**	**294/302**	**244/256**	**318/322**	**225/241**	**287/287**
		**KL**	**238**	**26-Jul-02**	**neg**						**2**	**141/161**	**196/196**	**186/190**	**294/302**	**244/256**	**318/322**	**225/241**	**287/287**
		**KL**	**245**	**21-Aug-02**	**neg**						**2**	**141/161**	**196/196**	**186/190**	**294/302**				
**Ch-086**	**F**	**KL**	**227**	**19-Jun-02**	**pos**	**pos**		**GU992204**		**TAN7**	**2**	**161/173**	**231/231**	**182/182**	**286/294**	**260/268**	**310/322**	**229/241**	**283/295**
		**KL**	**230**	**19-Jun-02**	**pos**	**neg**					**2**	**161/173**	**231/231**	**182/182**	**286/294**	**260/268**	**310/322**	**229/241**	**283/295**
		**KL**	**248**	**16-Aug-02**	**pos**	**neg**					**2**	**161/173**	**231/231**	**182/182**	**286/294**	**260/268**	**310/322**	**229/241**	**283/295**
		**KL**	**282**	**10-Jan-03**	**pos**	**neg**					**2**	**161/173**	**231/231**	**182/182**	**286/294**	**260/268**	**310/322**	**229/241**	**283/295**
		**KL**	**521**	**19-Jul-04**	**pos**	**neg**					**2**	**161/173**	**231/231**	**182/182**	**286/294**	**260/268**	**310/322**	**229/241**	**283/295**
		**KL**	**522**	**19-Jul-04**	**pos**	**neg**					**2**	**161/173**	**231/231**	**182/182**	**286/294**	**260/268**	**310/322**	**229/241**	**283/295**

Individual[1]	Sex[2]	Comm-unity[3]	Sample Code	Collection Date[4]	SIVcpz fecal WB[5]	SIVcpz fecal vRNA	Genbank Accession numbers[6] Full Length	pol	env	SIVcpz strain	mtDNA haplotype[7]	Microsatellite Loci D18s536	D4s243	D10s676	D9s922	D2s1326	D2s1333	D4s1627	D9s905
Ch-086 (cont)		KL	528	27-Jul-04	pos	neg					2	161/173	231/231	182/182	286/294	260/268	310/322	229/241	283/295
		KL	604	30-Jul-04	pos	neg					2	161/173	231/231	182/182	286/294	260/268	310/322	229/241	283/295
		KL	531	2-Aug-04	pos	neg					2	161/173	231/231	182/182	286/294	260/268	310/322	229/241	283/295
		KL	532	2-Aug-04	pos	neg					2	161/173	231/231	182/182	286/294	260/268	310/322	229/241	283/295
		KL	534	4-Aug-04	pos	neg					2	161/173	231/231	182/182	286/294	260/268	310/322	229/241	283/295
		KL	542	11-Aug-04	pos	neg					2	161/173	231/231	182/182	286/294	260/268	310/322	229/241	283/295
		KL	548	15-Aug-04	pos	neg					2	161/173	231/231	182/182	286/294	260/268	310/322	229/241	283/295
		KL	549	15-Aug-04	pos	neg					2	161/173	231/231	182/182	286/294	260/268	310/322	229/241	283/295
		KL	551	16-Aug-04	pos	pos					2	161/173	231/231	182/182	286/294	260/268	310/322	229/241	283/295
		KL	567	12-Sep-04	pos	neg					2	161/173	231/231	182/182	286/294	260/268	310/322	229/241	283/295
		KL	572	21-Sep-04	pos	neg					2	161/173	231/231	182/182	286/294	260/268	310/322	229/241	283/295
		KL	573	22-Sep-04	pos	neg					2	161/173	231/231	182/182	286/294	260/268	310/322	229/241	283/295
		KL	582	4-Oct-04	pos	neg					2	161/173	231/231	182/182	286/294	260/268	310/322	229/241	283/295
		KL	583	9-Oct-04	pos	neg					2	161/173	231/231	182/182	286/294	260/268	310/322	229/241	283/295
		KL	589	6-Nov-04	pos	neg					2	161/173	231/231	182/182	286/294	260/268	310/322	229/241	283/295
		KL	602	11-Dec-04	pos	neg					2	161/173	231/231	182/182	286/294	260/268	310/322	229/241	283/295
		KL	603	15-Dec-04	pos	neg					2	161/173	231/231	182/182	286/294	260/268	310/322	229/241	283/295
		KL	915	29-Dec-04	pos						2	161/173	231/231	182/182	286/294				
		KL	917	29-Dec-04	pos						2	161/173	231/231	182/182	286/294				
		KL	916	3-Jan-05	pos						2	161/173	231/231	182/182	286/294				
		KL	768	3-Mar-05	pos						2	161/173	231/231	182/182	286/294				
		KL	772	14-Mar-05	pos						2	161/173	231/231	182/182	286/294				
		KL	869	17-Jun-05	pos						2	161/173	231/231	182/182	286/294				
		KL	872	17-Jun-05	pos						2	161/173	231/231	182/182	286/294				
		KL	873	17-Jun-05	pos						2	161/173	231/231	182/182	286/294				
		KL	897	18-Sep-05	pos						2	161/173	231/231	182/182	286/294				
		KL	898	18-Sep-05	pos						2	161/173	231/231	182/182	286/294				
		KL	929	20-Nov-05	pos						2	161/173	231/231	182/182	286/294				
		KL	922	14-Jan-06	pos						2	161/173	231/231	182/182	286/294				
		KL	927	15-Jan-06	pos						2	161/173	231/231	182/182	286/294				
		KL	930	26-Jan-06	pos						2	161/173	231/231	182/182	286/294				
		KL	938	20-Feb-06	pos						2	161/173	231/231	182/182	286/294				
		KL	999	15-Mar-06	pos						2	161/173	231/231	182/182	286/294	260/268	310/322	229/241	283/295
		KL	1139	8-Aug-06	pos						2	161/173	231/231	182/182	286/294				
		KL	1177	11-Mar-07	pos						2	161/173	231/231	182/182	286/294				
		KL	1178	11-Mar-07	pos						2	161/173	231/231	182/182	286/294				
		KL	1180	12-Mar-07	pos						2	161/173	231/231	182/182	286/294				
		KL	1181	12-Mar-07	pos						2	161/173	231/231	182/182	286/294				
		KL	1284	7-Aug-07	pos							161/173	231/231	182/182	286/294				
		KL	1285	8-Aug-07	pos							161/173	231/231	182/182	286/294				
		KL	1286	8-Aug-07	pos							161/173	231/231	182/182	286/294				
		KL	1300	22-Aug-07	pos							161/173	231/231	182/182	286/294				
		KL	1394	22-Oct-07	pos							161/173	231/231	182/182	286/294				
		KL	1409	9-May-08	pos							161/173	231/231	182/182	286/294				
		KL	1470	14-Sep-08	pos							161/173	231/231	182/182	286/294				
		KL	1432	15-Sep-08	pos							161/173	231/231	182/182	286/294	260/268	310/322	229/241	283/295
		KL	1553	16-Apr-09	pos						2	161/173	231/231	182/182	286/294				
		KL	1554	16-Apr-09	pos						2	161/173	231/231	182/182	286/294				
		KL	1582	16-Jun-09	pos						2	161/173	231/231	182/182	286/294				
		KL	1583	16-Jun-09	pos						2	161/173	231/231	182/182	286/294				
		KL	1588	25-Jun-09	pos						2	161/173	231/231	182/182	286/294				
		KL	1695	11-Aug-09	pos						2	161/173	231/231	182/182	286/294				
		KL	1645	15-Sep-09	pos						2	161/173	231/231	182/182	286/294				
		KL	1779	12-Oct-09	pos						2	161/173	231/231	182/182	286/294				
		KL	1613	18-Oct-09	pos							161/173	231/231	182/182	286/294				
		KL	1751	18-Oct-09	pos						2	161/173	231/231	182/182	286/294				
		KL	911	na	pos						2	161/173	231/231	182/182	286/294				
Ch-087	M	KL	226	14-Jun-02	neg						2	141/141	196/204	190/190	294/294	256/260	310/322	229/241	287/295
Ch-088	F	KL	363	10-May-04	neg						2	141/173	196/231	158/182	294/294				
		KL	524	24-Jul-04	neg						2	141/173	196/231	158/182	294/294				
		KL	919	30-Dec-04	neg							141/173	196/231	158/182	294/294				
		KL	925	30-Dec-04	neg							141/173	196/231	158/182	294/294				
		KL	879	17-Jul-05	neg						2	141/173	196/231	158/182	294/294				
		KL	895	19-Aug-05	neg							141/173	196/231	158/182	294/294				
		KL	900	18-Sep-05	neg							141/173	196/231	158/182	294/294				
		KL	926	15-Jan-06	neg							141/173	196/231	158/182	294/294				
		KL	1138	6-Aug-06	neg							141/173	196/231	158/182	294/294				
		KL	1175	10-Mar-07	neg							141/173	196/231	158/182	294/294				
		KL	1305	6-Aug-07	neg							141/173	196/231	158/182	294/294				
		KL	1304[8]	28-Aug-07	neg							141/173	196/231	158/182	294/294	260/268	302/310	237/241	295/295
		KL	1524	6-Aug-08	neg							141/173	196/231	158/182	294/294	260/268	302/310	237/241	295/295
		KL	1528	6-Aug-08	neg							141/173	196/231	158/182	294/294	260/268	302/310	237/241	295/295
		KL	1587	25-Jun-09	neg						2	141/173	196/231	158/182	294/294	260/268	302/310	237/241	295/295
		KL	1730	11-Oct-09	neg							141/173	196/231	158/182	294/294	260/268	302/310	237/241	295/295
Ch-089	F	KL	237	15-Aug-02	neg						3	141/177	204/235	182/182	294/298				
		KL	170	23-Aug-02	neg						3	141/177	204/235	182/182	294/298				
		KL	240	23-Aug-02	neg						3	141/177	204/235	182/182	294/298	232/256	322/330	233/249	271/287
		KL	882	25-Jul-05	pos						3	141/177	204/235						
		KL	944	26-Apr-06	pos	pos		*FJ895396*		TAN18	3	141/177	204/235	182/182	294/298				
		KL	1303	25-Apr-07	pos							141/177	204/235	182/182	294/298	232/256	322/330	233/249	271/287
		KL	1277	6-Aug-07	pos							141/177	204/235	182/182	294/298	232/256	322/330	233/249	271/287
Ch-091	F	KL	364	5-Jun-04	pos	neg					10	141/161	196/196	186/190	290/302	244/248	318/322	225/241	279/287
Ch-092	F	KL	271	2-Mar-03	neg						11	145/149	200/204	178/178	298/302	260/264	310/322	241/249	287/295
Ch-093	F	KL	303	26-Apr-03	neg						11	153/161	200/204	182/190	268/286				
		KL	320	13-May-03	neg						11	153/161	200/204	182/190	268/286				
		KL	312	21-Aug-03	neg						11	153/161	200/204	182/190	268/286				
		KL	300	9-Oct-03	neg						11	153/161	200/204	182/190	268/286				
		KL	362	21-Dec-03	neg						11	153/161	200/204	182/190	268/286				
		KL	366	13-Jul-04	neg						11	153/161	200/204	182/190	268/286				
		KL	525	26-Jul-04	neg						11	153/161	200/204	182/190	268/286				
		KL	527	26-Jul-04	neg						11	153/161	200/204	182/190	268/286				
		KL	530	30-Jul-04	neg						11	153/161	200/204	182/190	268/286				
		KL	535	4-Aug-04	neg						11	153/161	200/204	182/190	268/286				
		KL	537	5-Aug-04	neg						11	153/161	200/204	182/190	268/286				
		KL	539	6-Aug-04	neg						11	153/161	200/204	182/190	268/286				
		KL	543	11-Aug-04	neg						11	153/161	200/204	182/190	268/286				
		KL	547A	13-Aug-04	neg						11	153/161	200/204	182/190	268/286				
		KL	552	26-Aug-04	neg						11	153/161	200/204	182/190	268/286				
		KL	571	21-Sep-04	neg						11	153/161	200/204	182/190	268/286				
		KL	578	4-Oct-04	neg						11	153/161	200/204	182/190	268/286				
		KL	581	4-Oct-04	neg						11	153/161	200/204	182/190	268/286				
		KL	590	6-Nov-04	neg						11	153/161	200/204	182/190	268/286				
		KL	591	6-Nov-04	neg						11	153/161	200/204	182/190	268/286				
		KL	600	7-Dec-04	neg						11	153/161	200/204	182/190	268/286				
		KL	767	13-Jan-05	neg							153/161	200/204	182/190	268/286				
		KL	780	29-Mar-05	neg							153/161	200/204	182/190	268/286				
		KL	871	17-Jun-05	neg							153/161	200/204	182/190	268/286				

							Genbank Accession numbers[6]					Microsatellite Loci							
Individual[1]	Sex[2]	Comm-unity[3]	Sample Code	Collection Date[4]	SIVcpz fecal WB[5]	SIVcpz fecal vRNA	Full Length	pol	env	SIVcpz strain	mtDNA haplotype[7]	D18s536	D4s243	D10s676	D9s922	D2s1326	D2s1333	D4s1627	D9s905
Ch-093 (cont)		KL	875	20-Jun-05	neg							153/161	200/204	182/190	268/286				
		KL	876	22-Jun-05	neg							153/161	200/204	182/190	268/286				
		KL	877	2-Jul-05	neg							153/161	200/204	182/190	268/286				
		KL	878	2-Jul-05	neg							153/161	200/204	182/190	268/286				
		KL	887	13-Aug-05	neg							153/161	200/204	182/190	268/286				
		KL	891	16-Aug-05	neg							153/161	200/204	182/190	268/286				
		KL	892	16-Aug-05	neg							153/161	200/204	182/190	268/286				
		KL	896	19-Sep-05	neg							153/161	200/204	182/190	268/286				
		KL	928	19-Jan-06	neg							153/161	200/204	182/190	268/286				
		KL	1128	6-Jun-06	neg							153/161	200/204	182/190	268/286				
		KL	1132	8-Jul-06	neg							153/161	200/204	182/190	268/286				
		KL	1137	22-Jul-06	neg							153/161	200/204	182/190	268/286				
		KL	1142	12-Aug-06	neg							153/161	200/204	182/190	268/286				
		KL	1145	29-Aug-06	neg							153/161	200/204	182/190	268/286				
		KL	1147	29-Aug-06	neg							153/161	200/204	182/190	268/286				
		KL	1204	11-Apr-07	neg							153/161	200/204	182/190	268/286				
		KL	1294	12-Aug-07	neg							153/161	200/204	182/190	268/286				
		KL	1295	12-Aug-07	neg							153/161	200/204	182/190	268/286				
		KL	1296	12-Aug-07	neg							153/161	200/204	182/190	268/286				
		KL	1290	9-Sep-07	neg							153/161	200/204	182/190	268/286				
		KL	1293	9-Sep-07	neg							153/161	200/204	182/190	268/286				
		KL	1297	1-Oct-07	neg							153/161	200/204	182/190	268/286				
		KL	1363	11-Dec-07	neg						11	153/161	200/204	182/190	268/286				
		KL	1339	14-Mar-08	neg							153/161	200/204	182/190	268/286				
		KL	1447	29-Jul-08	neg							153/161	200/204	182/190	268/286				
		KL	1450	29-Jul-08	neg							153/161	200/204	182/190	268/286				
		KL	1456	5-Aug-08	neg							153/161	200/204	182/190	268/286				
		KL	1522	11-Aug-08	neg							153/161	200/204	182/190	268/286				
		KL	1529	17-Sep-08	neg							153/161	200/204	182/190	268/286	260/264	310/322	241/249	287/295
		KL	1525	29-Nov-08	neg							153/161	200/204	182/190	268/286	260/264	310/322	241/249	287/295
		KL	1635	20-Jul-09	neg							153/161	200/204	182/190	268/286	260/264	310/322	241/249	287/295
		KL	1685	20-Jul-09	neg							153/161	200/204	182/190	268/286	260/264		241/249	287/295
		KL	1735	27-Jul-09	neg							153/161	200/204	182/190	268/286	260/264	310/322	241/249	287/295
		KL	1619	28-Jul-09	neg							153/161	200/204	182/190	268/286	260/264	310/322	241/249	287/295
		KL	1596	4-Aug-09	neg							153/161	200/204	182/190	268/286	260/264	310/322	241/249	287/295
		KL	1614	13-Aug-09	neg							153/161	200/204	182/190	268/286	260/264	310/322	241/249	287/295
		KL	1731	7-Oct-09	neg						11	153/161	200/204	182/190	268/286				
		KL	1681	14-Oct-09	neg							153/161	200/204	182/190	268/286	260/264		241/249	287/295
		KL	1722	14-Dec-09	neg							153/161	200/204	182/190	268/286			241/249	287/295
		KL	1737	22-Dec-09	neg							153/161	200/204	182/190	268/286	260/264	310/322	241/249	287/295
Ch-095	F	KL	541	11-Aug-04	neg						3	141/173	200/204	186/186	302/306				
		KL	544	11-Aug-04	neg						3	141/173	200/204	186/186	302/306				
		KL	545	11-Aug-04	neg						3	141/173	200/204	186/186	302/306				
		KL	559	30-Aug-04	neg						3	141/173	200/204	186/186	302/306				
		KL	560	30-Aug-04	neg						3	141/173	200/204	186/186	302/306				
		KL	566	30-Aug-04	neg						3	141/173	200/204	186/186	302/306	248/260	310/330	241/249	283/295
Ch-098	F	KL	249	14-Jun-02	neg						8	141/157	235/235	182/190	298/302				
		KL	250	14-Jun-02	neg						8	141/157	235/235	182/190	298/302				
		KL	235	26-Jun-02	neg						8	141/157	235/235	182/190	298/302				
		KL	318	29-Mar-03	neg						8	141/157	235/235	182/190	298/302				
		KL	321	30-Apr-03	neg						8	141/157	235/235	182/190	298/302				
		KL	323	30-Apr-03	neg						8	141/157	235/235	182/190	298/302				
		KK	765	21-Mar-05	neg						8	141/157	235/235	182/190	298/302	256/260	302/318	237/241	287/295
		MT	764	8-Apr-05	neg														
		MT	836	21-Aug-05	neg						8								
		MT	835	2-Sep-05	neg						8	141/157	235/235	182/190	298/302				
		MT	837	18-Oct-05	neg						8	141/157	235/235	182/190	298/302				
		MT	1077	20-Mar-06	neg						8	141/157	235/235	182/190	298/302				
		MT	1076	23-May-06	neg														
		MT	1203	7-Apr-07	neg						8	141/157	235/235	182/190	298/302				
		MT	1266	13-Aug-07	neg														
		MT	1265	1-Oct-07	neg														
		MT	1267	24-Jan-08	neg														
		MT	1268	24-Jan-08	neg							141/157	235/235	182/190	298/302				
		MT	1349	8-Jun-08	neg							141/157	235/235	182/190	298/302				
		MT	1593	30-Jul-09	neg									182/190	298/302			237/241	287/295
		MT	1686	23-Sep-09	neg							141/157	235/235	182/190	298/302	256/260	302/318		287/295
		MT	1711	1-Oct-09	neg							141/157	235/235	182/190	298/302	256/260	302/318	237/241	287/295
		MT	1726	3-Nov-09	neg							141/157	235/235	182/190	298/302	256/260	302/318	237/241	287/295
		MT	1687	na	neg						8	141/157	235/235	182/190	298/302	256/260	302/318	237/241	287/295
Ch-099	F	KL	299	17-Aug-03	pos	neg					10	141/171	196/196	186/190	290/298				
		KL	314	17-Aug-03	pos	neg					10	141/171	196/196	186/190	290/298				
		KL	316	21-Aug-03	pos	neg					10	141/171	196/196	186/190	290/298				
		KK	574	2-Oct-04	pos	pos					10	141/171	196/196	186/190	290/298	248/248	322/322	225/241	271/279
		KK	575	2-Oct-04	pos	pos					10	141/171	196/196	186/190	290/298	248/248	322/322	225/241	271/279
		KK	576	2-Oct-04	pos	pos					10	141/171	196/196	186/190	290/298	248/248	322/322	225/241	271/279
		KK	959	23-Dec-04	pos							141/171	196/196	186/190	290/298				
		KK	715	11-May-05	pos						10	141/171	196/196	186/190	290/298	248/248	322/322	225/241	271/279
		KK	783	12-Jul-05	pos	pos		*FJ895395*	*FJ895383*	TAN6	10	141/171	196/196	186/190	290/298				
		KK	853	25-Oct-05	pos							141/171	196/196	186/190	290/298				
		KK	958	15-Mar-06	pos							141/171	196/196	186/190	290/298				
Ch-100	M	KL	523	20-Jul-04	neg						13	141/161	196/200	190/190	298/302				
		KL	540	8-Aug-04	neg						13	141/161	196/200	190/190	298/302				
		KL	558	30-Aug-04	neg						13	141/161	196/200	190/190	298/302				
		KL	585	10-Oct-04	neg						13	141/161	196/200	190/190	298/302				
		KL	588	13-Oct-04	neg						13	141/161	196/200	190/190	298/302	252/256	322/322	237/241	287/295
		KL	924	30-Dec-04	neg							141/161	196/200	190/190	298/302				
		KL	779	29-Mar-05	neg							141/161	196/200	190/190	298/302				
		KL	950	17-Nov-05	neg							141/161	196/200	190/190	298/302				
		KL	936	5-Feb-06	neg							141/161	196/200	190/190	298/302				
		KL	937	5-Feb-06	neg							141/161	196/200	190/190	298/302				
		KL	948	17-Apr-06	neg							141/161	196/200	190/190	298/302				
		KL	946	17-May-06	neg							141/161	196/200	190/190	298/302				
		KL	1144	13-Aug-06	neg							141/161	196/200	190/190	298/302				
		KL	1361	28-Jun-07	neg						13	141/161	196/200	190/190	298/302				
		KL	1278	6-Dec-07	pos	pos		GU992205		TAN21		141/161	196/200	190/190	298/302				
		KL	1279	6-Dec-07	pos	pos			*FJ895392*	TAN21		141/161	196/200	190/190	298/302				
		KL	1487	17-Jul-08	pos	pos						141/161	196/200	190/190	298/302				
		KL	1531	9-Aug-08	pos	pos						141/161	196/200	190/190	298/302	252/256	322/322	237/241	287/295
		KL	1584	16-Jun-09	pos						13	141/161	196/200	190/190	298/302				
		KL	1606	20-Jul-09	pos							141/161	196/200	190/190	298/302				
		KL	1696	28-Jul-09	pos							141/161	196/200	190/190	298/302				
Ch-106	F	KL	1140	10-Aug-06	pos	pos		*FJ895401*		TAN20	9	141/177	196/204	182/190					
		KL	1425	5-Nov-07	pos							141/177	196/204	182/190	298/302				
		KL	1542	19-Jan-09	pos						9	141/177	196/204	182/190	298/302	236/256	310/322	233/237	295/295
		KL	901	na	pos	pos			*FJ895390*	TAN20	9	141/177	196/204	182/190	298/302	236/256	310/322	233/237	295/295
Ch-107	F	KL	889	13-Aug-05	pos	neg					2	161/173	196/231	154/178	286/294	260/268	310/322	229/241	283/295

Individual[1]	Sex[2]	Comm-unity[3]	Sample Code	Collection Date[4]	SIVcpz fecal WB[5]	SIVcpz fecal vRNA	Genbank Accession numbers[6]			SIVcpz strain	mtDNA haplotype[7]	Microsatellite Loci							
							Full Length	pol	env			D18s536	D4s243	D10s676	D9s922	D2s1326	D2s1333	D4s1627	D9s905
Ch-107 (cont)		KL	949	17-Nov-05	pos	neg					2	161/173	196/231	154/178	286/294	260/268	310/322	229/241	283/295
Ch-108[9]	F	KL	868	6-Jun-05	neg						3	141/177	204/231	182/190	290/298				
		KL	881	25-Jul-05	neg						3	141/177	204/231	182/190	290/298				
		KL	1000	16-Mar-06	neg						3	141/177	204/231	182/190	290/298	256/260	318/330	241/249	271/287
		KL	1090	26-Apr-06	neg							141/177	204/231	182/190	290/298				
		KL	1601	21-Jul-09	neg							141/177	204/231	182/190	290/298	256/260	318/330	241/249	271/287
Ch-109	F	KL	893	18-Aug-05	neg						13	141/161	200/200	182/190	286/302				
		KL	1348	12-Mar-08	neg						13	141/161	200/200	182/190	286/302	256/256	302/318	237/241	279/295
		KL	1462	14-Oct-08	neg							141/161	200/200	182/190	286/302	256/256	302/318	237/241	279/295
		KL	1705	9-Jul-09	neg							141/161	200/200	182/190	286/302	256/256	302/318	237/241	279/295
Ch-110	M	KL	939	13-Feb-06	neg						11	141/153	196/200	182/190	268/294				
		KL	1141	12-Aug-06	neg						11	141/153	196/200	182/190	268/294				
		KL	1418	11-Dec-07	neg							141/153	196/200	182/190	268/294				
		KL	1413	15-Jan-08	neg							141/153	196/200	182/190	268/294				
		KL	1338	12-Mar-08	neg							141/153	196/200	182/190	268/294	244/264	318/322	237/241	287/295
		KL	1463	28-Nov-08	neg							141/153	196/200	182/190	268/294	244/264	318/322	237/241	287/295
		KL	1633	20-Jul-09	neg							141/153	196/200	182/190	268/294	244/264	318/322	237/241	287/295
		KL	1734	28-Jul-09	neg							141/153	196/200	182/190	268/294	244/264	318/322	237/241	287/295
		KL	1683	13-Aug-09	neg							141/153	196/200	182/190	268/294	244/264	318/322	237/241	287/295
		KL	1736	22-Dec-09	neg							141/153	196/200	182/190	268/294	244/264	318/322	237/241	287/295
Ch-118	M	KL	1464	14-Oct-08	pos	neg						141/177	231/235	182/190	290/298	256/256	322/322	241/249	271/295
Ch-121	F	KL	1662	22-Nov-09	pos	neg					13	141/161	200/235	182/190	286/302				279/283

[1]Black, uninfected; red, SIVcpz infected. Some results in this table have been reported previously [2], but are included for completion; new data are highlighted in bold face.
[2]F, female; M, male.
[3]Resident community of the chimpanzee. KL, Kalande; MT, Mitumba; KK, Kasekela.
[4]na, not available.
[5]pos, positive; neg, negative.
[6]Previously reported Genbank Accession numbers are italicized.
[7]Numbers indicate mtDNA haplotypes as previously reported [2].
[8]Sample 1304 from Ch-088 was previously misidentified to represent Ch-108 [2].
[9]Unlike previously reported [2], Ch-108 did not permanently emigrate to KK on 25-Mar-06.
