# Supplementary material for: Impact of Simian Immunodeficiency Virus Infection on Chimpanzee Population Dynamics
Source: PLoS Pathog. 2010 Sep 23;6(9):e1001116. doi: 10.1371/journal.ppat.1001116 (PMC2944804; doi:10.1371/journal.ppat.1001116)
Supplement: Table S2 — Non-invasive testing of Mitumba and Kasekela chimpanzees for SIVcpz infection. (0.07 MB PDF) [file ppat.1001116.s003.pdf]

Table S2. Non-invasive testing of Kasekela and Mitumba chimpanzees for SIVcpz infection.[1]

							Microsatellite Loci			
**Individual[2]**	**Sex[3]**	**Comm-unity[4]**	**Sample Code**	**Collection Date**	**SIVcpz fecal WB[5]**	**mtDNA haplotype[6]**	**D18s536**	**D4s243**	**D10s676**	**D9s922**
Ch-001	F	KK	1537	15-Feb-09	neg	1	141/161	204/235	182/190	286/302
		KK	1555	9-Apr-09	neg	1	141/161	204/235	182/190	286/302
		KK	1637	11-Oct-09	neg		141/161	204/235	182/190	286/302
Ch-002	F	KK	1533	18-Feb-09	neg	7	141/161	204/231	182/190	302/302
		KK	1781	18-Oct-09	neg		141/161	204/231	182/190	302/302
Ch-003	M	KK	1759	9-Oct-09	neg		141/157	204/204	186/190	270/294
Ch-004	M	KK	1650	10-Sep-09	pos	6	141/161	200/235	182/182	286/302
		KK	1713	2-Oct-09	pos	6	141/161	200/235	182/182	286/302
		KK	1654	19-Nov-09	pos	6	141/161	200/235	182/182	286/302
Ch-005	M	KK	1780	2-Oct-09	neg		141/157	196/200	182/194	
Ch-007	M	KK	1718	7-Sep-09	neg		141/141	196/235	158/190	298/298
		KK	1659	23-Nov-09	neg		141/141	196/235	158/190	298/298
Ch-009	F	KK	1559	2-Jul-09	neg	6	141/173	196/200	190/194	302/306
		KK	1743	3-Sep-09	neg		141/173	196/200	190/194	302/306
		KK	1679	23-Oct-09	neg		141/173	196/200	190/194	
Ch-010	M	KK	1534	15-Jan-09	neg	6	141/161	200/235	182/194	286/302
		KK	1631	2-Oct-09	neg	6				
Ch-012	F	KK	1545	20-Apr-09	neg	6	141/173	196/200	182/194	298/302
		KK	1717	21-Oct-09	neg		141/173	196/200	182/194	298/302
Ch-013	M	KK	1605	10-Sep-09	neg		141/173	200/204	182/194	302/302
		KK	1599	11-Sep-09	neg		141/173	200/204	182/194	302/302
		KK	1612	2-Oct-09	neg		141/173		182/194	
		KK	1752	7-Oct-09	neg		141/173	200/204	182/194	302/302
Ch-014	M	KK	1558	18-Jun-09	neg	6	141/161	196/200	186/190	290/306
		KK	1653	8-Dec-09	neg	6	141/161	196/200	186/190	290/306
Ch-015	F	KK	1536	27-Jan-09	neg	1	141/161	235/235	182/190	286/298
		KK	1535	15-Feb-09	neg	1	141/161	235/235	182/190	286/298
		KK	1604	4-Oct-09	neg		141/161	235/235	182/190	286/298
		KK	1693	22-Oct-09	neg		141/161	235/235	182/190	286/298
Ch-017	F	KK	1782	3-Sep-09	neg		141/173	200/204	182/194	302/306
		KK	1755	7-Sep-09	neg		141/173		182/194	302/306
		KK	1636	9-Oct-09	neg		141/173	200/204	182/194	
		KK	1741	??-??-09	neg	1	141/173	200/204	182/194	302/306
Ch-018	F	KK	1714	11-Oct-09	neg		141/161	235/235	190/190	286/302
Ch-019	F	KK	1547	22-Apr-09	neg	11	153/157	204/235	186/190	290/298
		KK	1657	9-Dec-09	neg		153/157	204/235	186/190	290/298
Ch-020	M	KK	1539	19-Jan-09	neg	12	141/141	196/231	182/190	290/302
		KK	1709	9-Oct-09	neg		141/141	196/231	182/190	290/302
Ch-021	F	KK	1602	12-Aug-09	pos		161/177	204/204	182/186	294/302
Ch-022	F	KK	1565	19-Jun-09	pos	7	157/161	196/196	182/182	286/294
		KK	1715	6-Oct-09	pos		157/161	196/196	182/182	286/294
Ch-023	M	KK	1567	15-May-09	neg	9	141/161	235/235	182/190	286/298
		KK	1594	10-Oct-09	neg		141/161	235/235	182/190	286/298
Ch-025	F	KK	1622	12-Aug-09	neg		157/173	231/231	186/190	286/302
		KK	1703	27-Sep-09	neg		157/173	231/231	186/190	286/302
		KK	1742	11-Oct-09	neg		157/173	231/231	186/190	286/302
Ch-026	F	KK	1544	16-Jan-09	neg	5	153/161	196/235	186/186	286/298
		KK	1548	15-Apr-09	neg	5	153/161	196/235	186/186	286/298
		KK	1571	18-May-09	neg	5	153/161	196/235	186/186	286/298
		KK	1758	12-Oct-09	neg		153/161	196/235	186/186	286/298
Ch-027	M	KK	1572	4-May-09	neg	5	141/161	196/200	186/186	286/290
		KK	1648	8-Sep-09	neg		141/161	196/200	186/186	286/290
		KK	1677	5-Dec-09	neg		141/161	196/200	186/186	
Ch-029	F	KK	1611	5-Aug-09	neg		141/161	196/196	182/190	294/302
		KK	1704	16-Oct-09	neg		141/161	196/196	182/190	294/302
Ch-031	F	KK	1543	22-Jan-09	neg	5	157/161	196/231	182/186	286/302
		KK	1574	8-Apr-09	neg	5	157/161	196/231	182/186	286/302
		KK	1575	17-Jun-09	neg	5	157/161	196/231	182/186	286/302
		KK	1763	12-Oct-09	neg		157/161	196/231	182/186	
Ch-032	F	KK	1577	14-May-09	neg	11	161/173	196/200	190/190	302/306
		KK	1702	16-Sep-09	neg		161/173	196/200	190/190	302/306
		KK	1638	9-Oct-09	neg		161/173	196/200	190/190	302/306
Ch-034	M	KK	1579	22-Jun-09	neg			200/204	190/194	268/302
		KK	1610	24-Oct-09	neg		169/173	200/204	190/194	268/302

Individual[2]	Sex[3]	Comm-unity[4]	Sample Code	Collection Date	SIVcpz fecal WB[5]	mtDNA haplotype[6]	Microsatellite Loci D18s536	D4s243	D10s676	D9s922
Ch-035	M	KK	1627	10-Oct-09	neg		153/161	204/235	182/186	286/298
Ch-039	F	MT	1778	19-Aug-09	pos	12	141/153	200/204	186/186	286/298
		MT	1626	4-Sep-09	pos	12	141/153	200/204	186/186	286/298
		MT	1764	3-Oct-09	pos	12	141/153	200/204	186/186	286/298
Ch-039		MT	1716	4-Oct-09	pos	12	141/153	200/204	186/186	286/298
		MT	1642	13-Oct-09	pos	12	141/153	200/204	186/186	286/298
Ch-042	F	MT	1667	14-Nov-09	neg	2	141/173	200/235	190/190	286/294
		MT	1666	??-??-09	neg		141/173		190/190	286/294
Ch-044	F	MT	1721	10-Aug-09	neg		157/173	231/235	186/190	286/302
		MT	1720	28-Oct-09	neg		157/173	231/235	186/190	286/302
		MT	1652	8-Nov-09	neg		157/173	231/235	186/190	286/302
Ch-046	F	MT	1733	8-Dec-09	neg		141/161	204/235	190/194	298/306
		MT	1671	10-Dec-09	neg		141/161	204/235	190/194	298/306
		MT	1710	??-??-09	neg		141/161	204/235	190/194	298/306
Ch-047	F	MT	1591	10-Aug-09	neg				182/190	302/302
		MT	1768	15-Sep-09	neg		141/161	196/204	182/190	302/302
		MT	1658	3-Dec-09	neg		141/161	196/204	182/190	302/302
		MT	1674	6-Dec-09	neg		141/161	196/204	182/190	302/302
Ch-048	M	MT	1568	10-Jul-09	pos	13	161/173	231/235	186/190	302/306
		MT	1697	30-Sep-09	pos	13	161/173	231/235	186/190	302/306
		MT	1747	7-Nov-09	pos	13	161/173	231/235	186/190	302/306
Ch-049	F	MT	1777	26-Aug-09	neg		141/153	196/235	190/190	298/302
		MT	1615	19-Oct-09	neg		141/153	196/235	190/190	298/302
		MT	1670	18-Nov-09	neg		141/153		190/190	298/302
		MT	1676	18-Dec-09	neg	11	141/153	196/235	190/190	
Ch-050	F	KK	1744	2-Oct-09	neg		141/141	200/204	186/186	302/306
Ch-051	M	KK	1560	26-Jun-09	neg	6	141/161	200/200	186/190	290/306
		KK	1746	28-Jun-09	neg		141/161	200/200	186/190	290/306
		KK	1762	11-Oct-09	neg		141/161	200/200	186/190	290/306
Ch-052	M	KK	1541	19-Feb-09	pos	3	141/173	200/204	186/194	
		KK	1551	8-Apr-09	pos	3	141/173	200/204	186/194	298/302
		KK	1760	11-Sep-09	pos	3	141/173	200/204	186/194	298/302
		KK	1753	2-Oct-09	pos	3	141/173	200/204	186/194	298/302
Ch-053	F	KK	1632	4-Oct-09	neg		141/173	204/204	182/190	
Ch-054	M	KK	1573	23-Jun-09	neg	5	141/157	231/235	158/186	286/298
		KK	1643	2-Oct-09	neg		141/157	231/235	158/186	286/298
Ch-055	F	KK	1688	10-Sep-09	neg	8	157/161	200/204	182/186	298/302
		KK	1655	21-Nov-09	neg	8		200/204		
Ch-056	M	KK	1651	3-Aug-09	neg		157/173	204/231	182/194	286/302
		KK	1630	11-Oct-09	neg		157/173	204/231	182/194	286/302
Ch-057	F	KK	1748	15-Oct-09	neg		141/161	231/235	190/190	286/302
Ch-058	M	KK	1694	11-Oct-09	neg		141/173	200/200	182/190	268/302
Ch-059	M	MT	1707	13-Aug-09	neg		141/141	196/235	186/190	298/306
		MT	1765	4-Sep-09	pos	11	141/141	196/235	186/190	298/306
		MT	1757	1-Oct-09	neg		141/141	196/235	186/190	298/306
		MT	1689	6-Nov-09	neg	11	141/141	196/235	186/190	298/306
		MT	1673	3-Dec-09	neg	11	141/141	196/235	186/190	298/306
Ch-060	M	KK	1578	1-Jul-09	neg	11	141/161	196/231	190/190	290/302
		KK	1761	5-Aug-09	neg		141/161		190/190	290/302
		KK	1617	3-Oct-09	neg		141/161	196/231	190/190	290/302
		KK	1644	8-Oct-09	neg		141/161	196/231	190/190	290/302
		KK	1603	??-??-09	neg		141/161	196/231	190/190	290/302
Ch-061	F	KK	1625	2-Sep-09	neg		141/141	196/204	182/186	290/306
		KK	1754	4-Oct-09	neg		141/141	196/204	182/186	290/306
Ch-063	M	MT	1607	13-Aug-09	neg		161/173	200/204	182/190	294/298
		MT	1725	29-Sep-09	neg		161/173	200/204	182/190	294/298
		MT	1628	13-Oct-09	neg		161/173	200/204	182/190	294/298
Ch-065	M	MT	1698	31-Aug-09	neg		141/141	231/235	190/190	302/306
		MT	1771	4-Sep-09	neg		141/141	231/235	190/190	
		MT	1783	3-Oct-09	neg		141/141	231/235	190/190	302/306
		MT	1684	5-Nov-09	neg	6	141/141			
		MT	1669	3-Dec-09	neg		141/141	231/235	190/190	302/306
Ch-066	F	MT	1773	15-Aug-09	neg		161/177	200/235	182/190	
		MT	1589	3-Oct-09	neg		161/177	200/235	182/190	306/306
		MT	1719	3-Nov-09	neg		161/177		182/190	306/306
		MT	1663	18-Dec-09	neg		161/177	200/235	182/190	306/306

							Microsatellite Loci			
Individual[2]	Sex[3]	Comm-unity[4]	Sample Code	Collection Date	SIVcpz fecal WB[5]	mtDNA haplotype[6]	D18s536	D4s243	D10s676	D9s922
Ch-067	M	MT	1732	15-Dec-09	neg		141/161	196/200	182/190	302/302
Ch-068	M	MT	1557	10-Jul-09	neg	6	141/161	200/235	182/194	268/306
		MT	1682	4-Oct-09	neg		141/161		182/194	268/306
Ch-073	F	KK	1708	8-Sep-09	neg		141/173	204/231	186/194	302/302
Ch-075	M	MT	1634	3-Sep-09	neg		141/161	200/235	182/182	302/306
		MT	1616	3-Nov-09	neg		141/161	200/235	182/182	
Ch-076	F	MT	1774	23-Oct-09	neg		141/153	200/204	182/182	
		MT	1660	8-Dec-09	neg		141/153	200/204	182/182	268/302
Ch-077	M	KK	1561	27-Jun-09	neg	1	141/161	204/204	182/186	286/294
		KK	1647	8-Oct-09	neg		141/161	204/204	182/186	286/294
		KK	1706	21-Oct-09	neg		141/161	204/204	182/186	286/294
Ch-078	F	KK	1556	2-Jul-09	neg	12	141/153	204/204	186/186	286/298
		KK	1624	5-Aug-09	neg		141/153	204/204	186/186	286/298
		KK	1620	9-Oct-09	neg		141/153	204/204	186/186	286/298
Ch-079	F	KK	1566	8-Jul-09	neg	13	141/141	204/235	190/190	290/302
		KK	1701	11-Oct-09	neg	13	141/141	204/235	190/190	
Ch-080	F	KK	1629	3-Oct-09	pos		141/157	204/204	182/186	298/298
Ch-090	M	KK	1623	16-Oct-09	neg		141/141	204/231	186/190	290/298
		KK	1749	??-??-09	neg		141/141	204/231	186/190	
Ch-094	F	MT	1656	15-Dec-09	neg		157/173	231/235	186/190	302/302
Ch-096	F	KK	1569	17-Jun-09	neg		153/161	200/200	182/182	268/298
		KK	1680	10-Oct-09	neg		153/161	200/200	182/182	268/298
		KK	1646	23-Oct-09	neg		153/161	200/200	182/182	268/298
Ch-098	F	MT	1593	30-Jul-09	neg				182/190	298/302
		MT	1686	23-Sep-09	neg		141/157	235/235	182/190	298/302
		MT	1711	1-Oct-09	neg		141/157	235/235	182/190	298/302
		MT	1726	3-Nov-09	neg		141/157	235/235	182/190	298/302
		MT	1687	??-??-09	neg	8	141/157	235/235	182/190	298/302
Ch-101	F	KK	1769	30-Jul-09	neg		161/177	196/196		290/302
Ch-102	F	KK	1580	18-Jun-09	neg	6	141/161	200/200	186/190	286/302
		KK	1691	23-Sep-09	neg	6	141/161	200/200	186/190	286/302
		KK	1609	??-??-09	neg	6	141/161	200/200	186/190	
Ch-103	F	KK	1546	29-Apr-09	neg	3	161/161	204/235	186/190	298/302
		KK	1640	18-Aug-09	neg	3	161/161	204/235	186/190	298/302
		KK	1598	25-Aug-09	pos	3	161/161	204/235	186/190	298/302
		KK	1675	2-Dec-09	neg	3	161/161	204/235	186/190	298/302
Ch-105	F	KK	1745	23-Sep-09	neg		141/141	235/235	158/182	298/302
		KK	1595	10-Oct-09	neg		141/141	235/235	158/182	298/302
Ch-111	F	MT	1639	??-??-09	neg		141/141	200/235	182/190	268/302
		MT	1772	??-??-09	neg		141/141	200/235	182/190	268/302
Ch-112	F	MT	1590	9-Oct-09	neg		141/173	235/235	190/190	306/306
		MT	1668	8-Dec-09	neg		141/173	235/235	190/190	306/306
Ch-113	M	MT	1661	25-Nov-09	neg		153/161	200/204	182/182	268/268
Ch-114	F	KK	1576	14-May-09	neg	11	141/173	196/235	182/190	286/306
		KK	1608	15-Oct-09	neg		141/173	196/235	182/190	
Ch-115	M	KK	1550	24-Apr-09	neg	3	141/161	204/204	186/190	302/306
		KK	1770	3-Oct-09	neg		141/161	204/204	186/190	302/306
Ch-116	M	KK	1540	8-Feb-09	neg	5	141/157	196/231	190/190	286/298
		KK	1750	12-Aug-09	neg		141/157	196/231	190/190	
		KK	1690	29-Sep-09	neg	5	141/157	196/231	190/190	286/298
		KK	1678	19-Oct-09	neg		141/157	196/231	190/190	286/298
		KK	1641	22-Oct-09	neg		141/157	196/231	190/190	286/298
Ch-117	F	KK	1538	20-Jan-09	neg	11	153/161	204/204	186/190	286/298
	F	KK	1564	16-Jun-09	neg	11	153/161	204/204	186/190	286/298
		KK	1664	8-Dec-09	neg		153/161	204/204	186/190	286/298
Ch-119	M	KK	1552	26-Mar-09	neg	4	141/141	196/235	182/182	290/298
Ch-120	F	KK	1570	12-Jun-09	neg	5	141/153	196/235		298/298
		KK	1700	15-Sep-09	neg	5	141/153	196/235	158/186	298/298
Ch-122		MT	1665	5-Dec-09	neg	1	161/177	235/235	182/190	306/306
Ch-123		KK	1775	1-Oct-09	neg	6	141/161	200/235	182/190	286/302

[1]Results of samples collected from 01-Jan-09 to 31-Dec-09 are shown. Results for all samples collected previously are reported in [2].

[2]Black, SIVcpz uninfected; red, SIVcpz infected.

[3]F, female; M, male.

[4]Resident community of the chimpanzee. MT, Mitumba; KK, Kasekela.

[5]pos, positive; neg, negative.

[6]Numbers indicate mtDNA haplotypes as previously reported [2].
