# Supplementary material for: Impact of Simian Immunodeficiency Virus Infection on Chimpanzee Population Dynamics
Source: PLoS Pathog. 2010 Sep 23;6(9):e1001116. doi: 10.1371/journal.ppat.1001116 (PMC2944804; doi:10.1371/journal.ppat.1001116)
Supplement: Table S3 — Kalande demographics. (0.07 MB PDF) [file ppat.1001116.s004.pdf]

Table S3. Kalande demographics.

ID[1]	Sex[2]	Mother ID[3]	Possible birth year	Year of departure from Kalande[4]	Kalande membership[5] 1998	1999	2000	2001	2002	2003	2004	2005	2006	2007	2008	2009	Cause of departure	Cause of death	Evidence for existence	Comments
KLAM1	M	unk.	1968	1998	KL?	D	D	D	D	D	D	D	D	D	D	D	Death	Killed by people? Disease?	Body found	Dead male found on Nyamagoma beach with hands and genitals cut off, Jul/Aug-98. [15]
Ch-033	F	unk.	1985	1998	KL	KK	KK	KK	KK	KK	KK	KK	KK	KK	KK	D	Emigration	Unknown	Visual identification and genotype	Emigrated to Kasekela by 6-Feb-98; last seen 02-March-08.
KLSM1	M	unk.	1988	1998	KL	D	D	D	D	D	D	D	D	D	D	D	Death	Intergroup Aggression	Visual identification	Attacked and probably killed in Aug-98 [45]
KLI1	unk	unk.	1996	1998	KL	D	D	D	D	D	D	D	D	D	D	D	Death	Intergroup Aggression	Visual identification	Killed by Kasekela males Oct-98 [45]
KLAF1	F	unk.	1968	1998/1999	KL?	KL?	D	D	D	D	D	D	D	D	D	D	Death?	Killed by people? Disease?	Verbal report	"2 dead females outside the Park (Ngerwe 1998/99), reported from villagers" [15]
KLAF2	F	unk.	1969	1998/1999	KL?	KL?	D	D	D	D	D	D	D	D	D	D	Death?	Killed by people? Disease?	Verbal report	"2 dead females outside the Park (Ngerwe 1998/99), reported from villagers" [15]
KLAF3	F	unk.	1969	1999	KL?	KL?	D	D	D	D	D	D	D	D	D	D	Death	Killed by people? Disease?	Body found	Decomposing female found on Bwavi beach in Feb-99. Cause of death not clear. Around the same time an infant was offered for sale in Kigoma. [15]
KLI2	unk	unk.	1997	1999	KL?	KL?	D	D	D	D	D	D	D	D	D	D	Death?	Killed by people?	Verbal report	Infant reported for sale in Kigoma market Feb-99, possible infant of mother on beach [15]
Ch-029	F	unk.	1978	2000	KL	KL	KL	KK	KK	KK	KK	KK	KK	KK	KK	KK	Emigration	Alive	Visual identification and genotype	Emigrated to Kasekela by 14-Aug-00.
Ch-022	F	unk.	1988	2000	KL?	KL?	KL?	KK	KK	KK	KK	KK	KK	KK	KK	KK	Emigration	Alive	Visual identification and genotype	Emigrated to Kasekela by 01-Oct-00.
Ch-076	F	unk.	1986	2001	KL?	KL?	KL?	KL?	MT	MT	MT	MT	MT	MT	MT	MT	Emigration	Alive	Visual identification and genotype	Emigrated to Mitumba by 28-May-01.
KLAM2	M	unk.	1962	2002	KL	KL	KL	KL	KL	D	D	D	D	D	D	D	Death	Disease	Visual identification	Thin old male, last seen during respiratory outbreak.
KLAF4	F	unk.	1972	2002	KL?	KL?	KL?	KL?	KL?	D	D	D	D	D	D	D	Death	Disease	Visual identification and body found	Unnamed adult female died during respiratory outbreak (date of death 10-Oct-02). Possibly Ch-070, Ch-082, or Ch-083.
Ch-087	M	unk.	1972	2002	KL	KL	KL	KL	KL	D	D	D	D	D	D	D	Death	Killed by people?	Visual identification and genotype	Probably killed by people (date of death 13-Aug-02). [15]
KLSM2	M	unk.	1988	2002	KL?	KL?	KL?	KL?	KL?	D	D	D	D	D	D	D	Death	Disease	Visual identification and body found	Adolescent male (~14 years old) died during respiratory outbreak (date of death 10-Oct-02). Possibly Ch-081.
Ch-021	F	Ch-089	1987	2002	KL	KL	KL	KL	KL	KK	KK	KK	KK	KK	KK	KK	Emigration	Disease	Visual identification and genotype	First seen in Kasekela 22-Jul-02; body found 05-Jan-10.
Ch-079	F	unk.	1990	2002	KL	KL	KL	KL	KL	KK	KK	KK	KK	KK	KK	KK	Emigration	Alive	Visual identification and genotype	First seen in Kasekela 02-Aug-02.
Ch-081	M	unk.	unk.	2002	KL	KL	KL	KL	KL	D	D	D	D	D	D	D	Death?	Unknown	Genotype	Possibly KLSM2.
Ch-085	M	unk.	unk.	2002	KL	KL	KL	KL	KL	D	D	D	D	D	D	D	Death	Disease	Genotype	Adult male last seen in 2002 weak and with severe diarrhea. Possibly KLAM2.
BB-089	unk	Ch-089	2000	2002	N	N	N	B?	KL	KL?	D	D	D	D	D	D	Death	Unknown	Visual identification	BB-089 seen with mother (who was identified by fecal sample) on 23-Aug-02.
Ch-070	F	unk.	unk.	2002	KL?	KL	KL	KL	KL	KL?	KL?	KL?	D	D	D	D	Death?	Unknown	Genotype	Possibly KLAF4.
Ch-082	F	unk.	unk.	2002	KL?	KL	KL	KL	KL	KL?	KL?	KL?	D	D	D	D	Death?	Unknown	Genotype	Possibly KLAF4.
Ch-083	F	unk.	unk.	2002	KL?	KL	KL	KL	KL	KL?	KL?	KL?	D	D	D	D	Death?	Unknown	Genotype	Possibly KLAF4.
Ch-099	F	unk.	1972	2003	KL	KL	KL	KL	KL	KL	KK	KK	KK	D	D	D	Emigration	Injury	Visual identification and genotype	Transferred to Kasekela 15-Sep-03.
Ch-105	F	unk.	1992	2003	KL	KL	KL	KL	KL	KL	KK	KK	KK	KK	KK	KK	Emigration	Alive	Visual identification and genotype	Transferred to Kasekela by Dec-03.
Ch-101	F	Ch-099	2000	2003	N	N	N	KL	KL	KL	KK	KK	KK	KK	KK	KK	Emigration	Alive	Visual identification and genotype	Transferred to Kasekela 15-Sep-03.
Ch-084	F	unk.	unk.	2003	KL?	KL?	KL	KL	KL	KL	KL?	KL?	KL?	D	D	D	Death?	Unknown	Genotype	
Ch-092	F	unk.	unk.	2003	KL?	KL?	KL	KL	KL	KL	KL?	KL?	KL?	D	D	D	Death?	Unknown	Genotype	
Ch-091	F	Ch-099	1987	2004	KL?	KL?	KL?	KL	KL	KL	KL	KL?	KL?	KL?	D	D	Death?	Unknown	Genotype	
Ch-095	F	unk.	unk.	2004	KL?	KL?	KL?	KL	KL	KL	KL	KL?	KL?	KL?	D	D	Death?	Unknown	Genotype	
Ch-098	F	unk.	1991	2004	KL	KL	KL	KL	KL	KL	KL	MT	MT	MT	MT	MT	Emigration	Alive	Visual identification and genotype	First seen in Mitumba 08-Jun-04.
Ch-071	F	Ch-099	1992	2005	KL	KL	KL	KL	KL	KL	KL	KL	KK	KK	KK	KK	Emigration	Alive	Visual identification and genotype	Transferred to Kasekela 15-Feb-05.
Ch-107	F	unk.	unk.	2005	KL?	KL?	KL?	KL?	KL	KL	KL	KL	KL?	KL?	KL?	D	Death?	Unknown	Genotype	
KLAF5	F	unk.	1979	NA	KL?	KL?	KL?	KL?	KL?	KL?	KL?	KL?	KL?	KL?	KL?	KL?	Possibly still present	Alive?	Verbal report	Possibly Ch-089, Ch-106, Ch-109, or Ch-121
KLSM3	M	KLAF5	1994	NA	KL?	KL?	KL?	KL?	KL?	KL?	KL?	KL?	KL?	KL?	KL?	KL?	Possibly still present	Alive?	Verbal report	Verbally reported sighting in Aug-05 and Jun-10
KLSF1	F	unk.	1988	NA	KL?	KL?	KL?	KL?	KL?	KL?	KL?	KL?	KL?	KL?	KL?	KL?	Possibly still present	Alive?	Verbal report	Seen in Kalande in 2005; may be female who visited Kasekela Feb-06.
KLSF2	F	KLAF4	1996	NA	KL?	KL?	KL?	KL?	KL?	KL?	KL?	KL?	KL?	KL?	KL?	KL?	Possibly still present	Alive?	Visual identification	Possibly Ch-106, Ch-109, or Ch-121
KLAM4	M	unk.	1978	NA	KL	KL	KL	KL	KL	KL	KL	KL	KL	KL	KL	KL	Likely still present	Alive	Visual identification	Adult male, regularly seen but apparently not yet sampled.
Ch-108	F	Ch-089	1993	2006	KL	KL	KL	KL	KL	KL	KL	KL	KL	KL	KL	KL	Likely still present	Alive	Visual identification and genotype	
Ch-064	F	unk.	1975	NA	KL	KL	KL	KL	KL	KL	KL	KL	KL	KL	KL	KL	Likely still present	Alive	Visual identification and genotype	
Ch-086	F	unk.	1985	NA	KL	KL	KL	KL	KL	KL	KL	KL	KL	KL	KL	KL	Likely still present	Alive	Visual identification and genotype	Regularly seen adult female.
Ch-089	F	unk.	1972	NA	KL	KL	KL	KL	KL	KK	KK	KL	KL	KL	KL	KL	Likely still present	Alive	Visual identification and genotype	

ID[1]	Sex[2]	Mother ID[3]	Possible birth year	Year of departure from Kalande[4]	Kalande membership[5] 1998	1999	2000	2001	2002	2003	2004	2005	2006	2007	2008	2009	Cause of departure	Cause of death	Evidence for existence	Comments
Ch-093	F	Ch-024	1984	NA	MT	KL	KL	KL	KL	KL	KL	KL	KL	KL	KL	KL	Likely still present	Alive	Visual identification and genotype	Likely transferred from Mitumba in Oct-98. Visited Kasekela Mar-06, Mar-08, but was back in Kalande by Aug-08.
Ch-088	F	Ch-086	2000	NA	N	N	N	N	B	KL	KL	KL	KL	KL	KL	KL	Likely still present	Alive	Visual identification and genotype	Seen as newborn 15-Sep-02.
Ch-100	M	Ch-064	2001	NA	N	N	N	B	KL	KL	KL	KL	KL	KL	KL	KL	Likely still present	Alive	Visual identification and genotype	
Ch-109	F	unk.	1999	NA	KL?	KL?	KL?	KL?	KL	KL	KL	KL	KL	KL	KL	KL	Likely still present	Alive	Genotype	Described as an adult female with sexual swelling when seen in 2005.
Ch-110	M	Ch-093	2002	NA	N	N	N	N	B	KL	KL	KL	KL	KL	KL	KL	Likely still present	Alive	Visual identification and genotype	Visited Kasekela Mar-06, Mar-08, but back in Kalande by Aug-08.
Ch-106	F	unk.	unk.	NA	KL?	KL?	KL?	KL?	KL?	KL	KL	KL	KL	KL	KL	KL	Likely still present	Alive	Genotype	
Ch-118	M	Ch-089	2005	NA	N	N	N	N	N	N	N	B	KL	KL	KL	KL	Likely still present	Alive	Genotype	Possibly KLSM3.
Ch-121	F	unk.		NA	KL?	KL?	KL?	KL?	KL?	KL?	KL?	KL?	KL	KL	KL	KL	Likely still present	Alive	Genotype	
BB-064	unk	Ch-064	2007	NA	N	N	N	N	N	N	N	N	N	B	KL	KL	Likely still present	Alive	Visual identification	BB-064 first seen 28-Jan-07 with its mother and was estimated to be 2-4 weeks old.
BB-093	unk	Ch-093	2009	NA	N	N	N	N	N	N	N	N	N	N	N	B	Likely still present	Alive	Visual identification	BB-093 was first seen 23-Jun-09.
				**Minimum population**	19	20	22	24	28	20	15	13	13	13	14	14				
				**Maximum population**	43	40	36	35	36	29	25	25	22	20	19	18				

[1]ID, chimpanzee identification; individuals identified from genotyped fecal samples are named following the convention Ch-###, while chimpanzees not linked to genotyped samples are named with a code that indicates community of residence (KL, Kalande), age class (A, adult, S, subadult, I, immature), sex (M, male, F, female), and a number indicating the order in which they were named.

[2]M, male; F, female; unk., unknown.

[3]Mother ID, identity of the mother; unk., unknown (see Methods and Table S5 for more information).

[4]The year of departure is based on the last sighting or sampling of the individual in Kalande or their date of arrival in another community; NA, not applicable.

[5]The columns indicate the status of each individual at the start of the year, including community membership (KL, Kalande, KK, Kasekela, MT, Mitumba) and life history status (N, not yet born ,B, born during that year, D, dead). Question marks indicate that the individual's status is uncertain for that year. Each cell is color-coded to indicate the quality of evidence for the existence and residence location of each individual during that year (no color, status uncertain; tan, verbal report; light yellow, visual observation; light green, inferred to be present; dark green, genotyped fecal sample collected; blue, known or presumed to have died or emigrated).
