# Supplementary material for: Impact of Simian Immunodeficiency Virus Infection on Chimpanzee Population Dynamics
Source: PLoS Pathog. 2010 Sep 23;6(9):e1001116. doi: 10.1371/journal.ppat.1001116 (PMC2944804; doi:10.1371/journal.ppat.1001116)
Supplement: Table S4 — Evidence for existence, departure, and causes of death for Kalande chimpanzees. (0.05 MB PDF) [file ppat.1001116.s005.pdf]

Table S4. Evidence for existence, departure, and causes of death for Kalande chimpanzees.

Evidence for existence	Number of individuals	SIVcpz negative	SIVcpz positive	SIVcpz status unknown	Individuals[1]
Visual identification AND genotype	20	12	8	0	Ch-021[2], Ch-022, Ch-029, Ch-033[2], Ch-064, Ch-071, Ch-076, Ch-079, Ch-086, Ch-087, Ch-088, Ch-089, Ch-093, Ch-098, Ch-099[2], Ch-100, Ch-101, Ch-105, Ch-108, Ch-110
Visual identification	8	unknown	unknown	8	BB-064, BB-089, KLAM2, KLAM4, KLI1, KLSM1, KLSF2, BB-093
Genotype	14	7	7	0	Ch-070, Ch-081, Ch-082, Ch-083, Ch-084, Ch-085, Ch-091, Ch-092, Ch-095, Ch-106, Ch-107, Ch-109, Ch-118, Ch-121
Verbal report	6	unknown	unknown	6	KLAF1, KLAF2, KLAF5, KLI2, KLSF1, KLSM3,
Visual identification AND body found	2	unknown	unknown	2	KLAF4, KLSM2
Body found	2	unknown	unknown	2	KLAF3, KLAM1
**Total**	**52**	**19**	**15**	**18**	
**Cause of departure**					
Emigration	11	7[2]	4	0	Ch-021[2], Ch-022, Ch-029, Ch-033[2], Ch-071, Ch-076, Ch-079, Ch-098, Ch-099[2], Ch-101, Ch-105
Death	10	2	0	8	BB-089, Ch-085, Ch-087, KLAF3, KLAF4, KLAM1, KLAM2, KLI1, KLSM1, KLSM2
Death?	12	5	4	3	Ch-070, Ch-081, Ch-082, Ch-083. Ch-084, Ch-091, Ch-092, Ch-095, Ch-107, KLAF1, KLAF2, KLI2
Possibly still present	4	unknown	unknown	4	KLAF5, KLSF1, KLSF2, KLSM3
Likely still present	15	5	7	3	BB-064, BB-093, Ch-064, Ch-086, Ch-088, Ch-089, Ch-093, Ch-100, Ch-106, Ch-108, Ch-109, Ch-110, Ch-118, Ch-121, KLAM4
**Total**	**52**	**19**	**15**	**18**	
**Cause of death**					
Unknown	11	5	5	1	Ch-033[2], Ch-70, Ch-081, Ch-082, Ch-083, Ch-084, Ch-091, Ch-092, Ch-095, Ch-107, BB-089
Disease	5	1	1	3	Ch-021[2], Ch-085, KLAF4, KLSM2, KLAM2
Killed by people? Disease?	4	0	0	4	KLAF1, KLAF2, KLAF3, KLAM1
Killed by people?	2	1	0	1	Ch-087, KLI2
Intergroup aggression	2	0	0	2	KLI1, KLSM1
Injury	1	0	1	0	Ch-099[2]
**Total**	**25**	**7**	**7**	**11**	

[1]Red, infected with SIVcpz; black, not infected with SIVcpz; blue, not tested for SIVcpz infection.
[2]Ch-021, Ch-033 and Ch-099 died after emigrating from Kalande.
