# Supplementary material for: Impact of Simian Immunodeficiency Virus Infection on Chimpanzee Population Dynamics
Source: PLoS Pathog. 2010 Sep 23;6(9):e1001116. doi: 10.1371/journal.ppat.1001116 (PMC2944804; doi:10.1371/journal.ppat.1001116)
Supplement: Table S5 — Genetic evidence for dyadic relationships. (0.11 MB PDF) [file ppat.1001116.s006.pdf]

Table S5. Genetic evidence of dyadic relatedness.

Target[1]	Possible Kin	MT Haplotype[2]	Number of Mismatches[3]	CERVUS Parentage Confidence Level[4]	KINSHIP Relatedness (R)[5]	KINSHIP Parent-Offspring[6]	KINSHIP Full Sibling[7]	KINSHIP Maternal Half Sibling[8]	KINSHIP Paternal Half Sibling[9]	Likely Relationship to Target
**Ch-047**	**Ch-082**	1	0	80%	0.37	*	NS	NS	NS	Mother or other close kin
**Ch-082**	**Ch-083**	1	1	NA	0.40	NA	*	*	*	Sibling
**Ch-083**	**Ch-047**	1	3	NA	0.04	NA	NS	NS	NS	None
Ch-081	*Ch-077*[10]	1	0	NA	0.38	NA	*	*	NS	None
**Ch-088**	**Ch-086**	2	0	80%	0.48	***	***	***	***	Mother
**Ch-088**	**Ch-107**	2	1	NA	0.42	NA	***	***	***	Other kin
**Ch-086**	**Ch-107**	2	1	NA	0.68	NA	***	***	***	Sibling
**Ch-088**	**Ch-029**	2	0	NS	0.46	*	*	*	*	Other kin
**Ch-029**	**Ch-107**	2	1	NA	0.12	NA	NS	NS	NS	None
**Ch-029**	**Ch-086**	2	1	NA	0.13	NA	NS	NS	NS	None
Ch-087	**Ch-029**	2	0	95%	0.39	*	*	*	*	Other kin
Ch-087	**Ch-088**	2	1	NA	0.32	NA	*	*	*	Other kin
Ch-087	**Ch-107**	2	2	NA	0.19	NA	*	*	*	Other kin
Ch-087	**Ch-086**	2	3	NA	0.06	NA	NS	NS	NS	None
Ch-087	*Ch-085*	2	0	95%[11]	0.38	*	*	*	*	Sibling or other kin
**Ch-029**	*Ch-085*	2	1	NA	0.45	NA	*	*	*	Sibling or other kin
**Ch-088**	*Ch-085*	2	4	NA	-0.11	NA	NS	NS	NS	None
**Ch-107**	*Ch-085*	2	3	NA	-0.03	NA	NS	NS	NS	None
**Ch-086**	*Ch-085*	2	4	NA	-0.22	NA	NS	NS	NS	None
**Ch-070**	**Ch-042**	2	1	NA	0.19	NA	NS	NS	NS	None
**Ch-070**	**Ch-029**	2	1	NA	0.18	NA	NS	NS	NS	None
**Ch-108**	**Ch-089**	3	0	95%	0.45	***	***	***	***	Mother
**Ch-021**	**Ch-089**	3	0	95%	0.41	***	***	***	***	Mother
**Ch-103**[12]	**Ch-089**	3	6	NA	0.24	NA	NS	NS	NS	Grandmother
Ch-118	**Ch-089**	3	0	95%	0.43	***	*	*	***	Mother
Ch-118	**Ch-108**	3	1	NA	0.46	NA	***	***	***	Sibling
**Ch-021**	**Ch-108**	3	3	NA	0.20	NA	NS	*	*	Sibling
Ch-118	**Ch-021**	3	4	NA	-0.17	NA	NS	NS	NS	Sibling
**Ch-095**	**Ch-050**[13]	3	0	95%	0.92	***	***	***	***	Sibling or other kin
Ch-052[13]	**Ch-095**	3	0	NA	0.50	NA	*	***	NS	Other kin
**Ch-061**[13]	**Ch-095**	3	0	NA	0.38	NA	*	***	NS	Other kin
**Ch-080**	**Ch-106**	9	1	NA	0.27	NA	NS	NS	NS	None
**Ch-101**	**Ch-099**	10	0	80%	0.59	***	***	***	***	Mother
**Ch-071**	**Ch-099**	10	0	80%	0.54	***	***	***	***	Mother
**Ch-091**	**Ch-099**	10	0	Ch-071[14] 80%	0.62	***	***	***	***	Mother

Target[1]	Possible Kin	MT Haplotype[2]	Number of Mismatches[3]	CERVUS Parentage Confidence Level[4]	KINSHIP Relatedness (R)[5]	KINSHIP Parent-Offspring[6]	KINSHIP Full Sibling[7]	KINSHIP Maternal Half Sibling[8]	KINSHIP Paternal Half Sibling[9]	Likely Relationship to Target
**Ch-091**	**Ch-071**	10	0	80%	0.75	***	***	***	***	Sibling
**Ch-101**	**Ch-091**	10	0	Ch-099[14] 80%	0.83	***	***	***	***	Sibling
**Ch-101**	**Ch-071**	10	2	NA	0.71	NA	***	***	***	Sibling
**Ch-093**	**Ch-024**[15]	11	0	95%	0.21	***	*	***	***	Mother
**Ch-093**	*Ch-034*[15]	11	2	NA	0.02	NA	*	***	NS	Sibling
**Ch-093**	*Ch-058*[15]	11	2	NA	0.06	NA	NS	*	NS	Sibling
Ch-110	**Ch-093**	11	0	95%	0.31	***	*	***	***	Mother
**Ch-092**	**Ch-093**	11	3	NA	0.36	NA	***	*	*	Other kin
Ch-110	**Ch-024**	11	1	NA	0.28	NA	*	*	***	Grandmother
**Ch-092**	**Ch-024**	11	2	NA	-0.01	NA	NS	*	*	Other kin
Ch-110	**Ch-092**	11	3	NA	-0.06	NA	NS	NS	NS	None
**Ch-033**	**Ch-093**	11	1	NA	0.23	NA	*	*	*	Other kin
**Ch-033**	**Ch-092**	11	2	NA	0.08	NA	NS	NS	NS	None
**Ch-033**	**Ch-024**	11	4	NA	-0.09	NA	NS	NS	NS	None
**Ch-033**	*Ch-110*	11	3	NA	-0.02	NA	NS	NS	NS	None
**Ch-079**	**Ch-064**	13	0	80%	0.36	*	*	*	*	Mother
Ch-100	**Ch-064**	13	0	80%	0.43	*	*	NS	NS	Mother or other close kin
Ch-100	**Ch-079**	13	2	NA	0.36	NA	NS	NS	NS	Sibling or other kin
**Ch-109**	**Ch-084**	13	0	95%	0.67	*	*	*	*	Sibling or other kin
**Ch-084**	*Ch-048*[16]	13	0	NS	0.09	NS	NS	NS	NS	None
**Ch-109**	*Ch-048*	13	2	NA	-0.13	NA	NS	NS	NS	None

[1]The most recent community of residence of the Target and Possible Kin chimpanzee is coded by color (Kalande, orange; Kasekela, brown; Mitumba, blue). Females are in bold while males are in non-bold italics.
[2]MT Haplotype, numbers indicate the mitochondrial DNA haplotype of both the Target and Possible Kin as previously reported [2].
[3]Number of Mismatches, number of microsatellite allelic mismatches between the Target/Possible Kin dyad.
[4]CERVUS Parentage Confidence Level, the relaxed confidence level from CERVUS (80%) and the strict confidence level (95%); most likely, the individual is the strongest candidate parent but did not fit the confidence level criteria; NS, CERVUS did not find a relationship between the dyad; NA, the pair was not tested as parent-offspring in CERVUS due to allelic mismatches or previously known parent-offspring relationships.
[5]KINSHIP Relatedness (R), the dyadic relatedness value for the Target and Possible Kin from KINSHIP. Values should be approximately 0.50 for parent-offspring and full siblings and 0.25 for half-siblings; however, values can vary considerably when examining small numbers of genetic regions.
[6]KINSHIP Parent-Offspring, significance from KINSHIP for the relationship; asterisks indicate p-value: *** $P < 0.001$; * $P \leq 0.05$; NA, the pair was not tested as parent-offspring in KINSHIP due to allelic mismatches or previously known parent-offspring relationships; NS, not significant.
[7]KINSHIP Full Sibling, significance level from KINSHIP for the relationship.
[8]KINSHIP Maternal Half Sibling, significance level from KINSHIP for the relationship.
[9]KINSHIP Paternal Half Sibling, significance level from KINSHIP for the relationship.
[10] Maternity and paternity are both known for Ch-077 and neither parent is Ch-081. Ch-077 was below reproductive age and was not considered as a potential parent to Ch-081.
[11]It is possible, but unlikely, for a father to be from within the same haplotype: of 39 known paternities to date, only two were between parents sharing the same haplotype (Wroblewski, unpublished data), and one of which was an unusual case of inbreeding [33].
[12]Ch-021 is the known mother to Ch-103.
[13]Ch-050 is the known mother to Ch-052 and Ch-061; neither Ch-052 nor Ch-061 were of age to be considered as potential parents to Ch-095.
[14]Parent for Target identified by CERVUS was not the Possible Kin.
[15]Ch-024 is the known mother to Ch-034 and Ch-058.
[16]Maternity was known for Ch-048 and it was not Ch-084.
