# Supplementary material for: Impact of Simian Immunodeficiency Virus Infection on Chimpanzee Population Dynamics
Source: PLoS Pathog. 2010 Sep 23;6(9):e1001116. doi: 10.1371/journal.ppat.1001116 (PMC2944804; doi:10.1371/journal.ppat.1001116)
Supplement: Table S6 — Genetic evidence for paternal relationships. (0.06 MB PDF) [file ppat.1001116.s007.pdf]

Table S6. Genetic evidence for paternal relationships.

Offspring[1]	Mother	Father	CERVUS Parentage Confidence Level[2]	KINSHIP Relatedness (R) (Father-Offspring)[3]	KINSHIP Parent-Offspring[4]
**Ch-088**	**Ch-086**	*Ch-081*	95%	0.36	*
Ch-118	**Ch-089**	*Ch-020*	95%	0.40	*
**Ch-101**	**Ch-099**	*Ch-085*	95%	0.61	*
**Ch-071**	**Ch-099**	[*Ch-085*][5,6]	[95%]	0.52	*
**Ch-091**	**Ch-099**	*Ch-085*	95%	0.74	***
**Ch-093**	**Ch-024**	*Evered*	80%	0.24	*
Ch-110	**Ch-093**	Ch-081	NS[7]	0.06	*
Ch-100	**Ch-064**	[*Ch-085*][5,8]	[most likely]	0.39	*

[1]The most recent community of residence of the Offspring, Mother, and Father chimpanzee is coded by color (Kalande, orange; Kasekela, brown; Mitumba, blue). Females are in bold while males are in non-bold italics.
[2]CERVUS Parentage Confidence Level, the relaxed confidence level from CERVUS (80%) and the strict confidence level (95%); most likely, the individual is the strongest candidate parent but did not fit the confidence level criteria; NS, not significant.
[3]KINSHIP Relatedness (R) (Father-Offspring), dyadic relatedness value for the Father and Offspring from KINSHIP. Values should be roughly 0.5 for parent-offspring; however, values can vary considerably when examining small numbers of genetic regions.
[4]KINSHIP Parent-Offspring, significance from KINSHIP for the relationship between father and offspring; asterisks indicate p-value: *** $P < 0.001$; * $P < 0.05$.
[5]There were no microsatellite allelic mismatches for the offspring/mother/father triad unless enclosed in brackets. In those cases, the confidence value, also in brackets, only applies when the mother is not included as the mother in CERVUS paternity analyses.
[6]When Ch-099 is included as the mother of Ch-071, no male lacks mismatches with the genotypes of Ch-099 and Ch-071.
[7]When Ch-093 was included as the mother of Ch-110, Ch-085 scored as the “most likely” father, but had one mismatch with the proposed mother-offspring pair. Ch-081 was the only male that lacked mismatches with the mother-offspring pair.
[8]When Ch-064 is included as the mother of Ch-100, no male lacks mismatches with the genotypes of Ch-064 and Ch-100.
